# Exploring the Connection of Brain Computer Interfaces and Multimedia Use With the Social Integration of People With Various Motor Disabilities: A Questionnaire-Based Usability Study

**DOI:** 10.3389/fdgth.2022.846963

**Published:** 2022-08-04

**Authors:** Ioulietta Lazarou, Spiros Nikolopoulos, Kostas Georgiadis, Vangelis P. Oikonomou, Agnes Mariakaki, Ioannis Kompatsiaris

**Affiliations:** ^1^Centre for Research and Technology Hellas (CERTH-ITI), Information Technologies Institute, Thessaloniki, Greece; ^2^Muscular Dystrophy Association-Hellas (MDA-Hellas), Hellas, Athens, Greece

**Keywords:** Brain Computer Interfaces, motor disabilities, social inclusion, Parkinson's disease, spinal cord injury, neuromuscular disorders

## Abstract

We have designed a platform to aid people with motor disabilities to be part of digital environments, in order to create digitally and socially inclusive activities that promote their quality of life. To evaluate in depth the impact of the platform on social inclusion indicators across patients with various motor disabilities, we constructed a questionnaire in which the following indicators were assessed: (i) Well Being, (ii) Empowerment, (iii) Participation, (iv) Social Capital, (v) Education, and (vi) Employment. In total 30 participants (10 with Neuromuscular Disorders-NMD, 10 with Spinal Cord Injury-SCI, and 10 with Parkinson's Disease-PD) used the platform for ~1 month, and its impact on social inclusion indicators was measured before and after the usage. Moreover, monitoring mechanisms were used to track computer usage as well as an online social activity. Finally, testimonials and experimenter input were collected to enrich the study with qualitative understanding. All participants were favorable to use the suggested platform, while they would prefer it for longer periods of time in order to become “re-awakened” to possibilities of expanded connection and inclusion, while it became clear that the platform has to offer them further the option to use it in a reclining position. The present study has clearly shown that the challenge of social inclusion cannot be tackled solely with technology and it needs to integrate persuasive design elements that foster experimentation and discovery.

## Introduction

Individuals who suffer from loss of the voluntary muscular control while preserving cognitive functions are marginalized and unable to keep up with the rest of the society in a digitized world. One of the most well-known conditions is the spinal cord injury (SCI), which is defined as damage of the spinal cord or nerves at the end of the spinal canal. Reasons for SCI may vary and can be divided into traumatic (e.g., sports etc.) and non-traumatic (e.g., medical condition). About 330,000 people with SCI are living in Europe, with 11,000 new injuries occurring per year (1, 2). These individuals lose their independence and privacy almost completely, which results in a tremendous decrease in quality of life (3). Another condition causing motor-impairment is the Parkinson's disease (PD), which is a common neurodegenerative disorder affecting ~1% of the population over the age of 60 years (4, 5), causing bradykinesia, tremor at rest, rigidity, postural instability, flexed posture and freezing. It has an age-adjusted frequency of 1.3 cases per 100,000 people younger than 45 years old and 3,100 for those of 75–85, respectively (6). PD has substantial implications for their social life, given that leisure activities that involve going out or relies on physical dexterity, can become difficult to maintain and lead to social isolation, while feelings of shame or stigma can result where a lack of social competence is perceived. Health related quality of life is severely compromised in PD in multiple domains and although disease specific disabilities are cardinal factors emotional and social aspects play an important role, too (7, 8). On the other hand, neuromuscular disorders (NMD) compromise a very broad term that encompasses many diseases and ailments that impair the functioning of the muscles, either directly, being pathologies of the muscle, or indirectly, being pathologies of nerves or neuromuscular junctions (9). In general, NMD can cause problems with central nervous control or some degree of paralysis, depending on the location and the nature of the problem. Spinal muscular atrophies are disorders of lower motor neuron while amyotrophic lateral sclerosis is a mixed upper and lower motor neuron condition. Muscular dystrophies and inflammatory myopathies are examples of primary muscular disorders, while the epidemiology and etiology of these diseases is very wide broad.

### The Importance of Technology for People With Motor Impairment

Interactive devices are used for enabling environmental control and computer, internet, and social media access to compensate the loss of motor function and to allow individuals with severe disabilities to participate in society (10). The social media access is extremely important for people with severe motor impairments, because in the virtual world, people with motor impairment are on the same level than non-impaired people. Nevertheless, joysticks for the hand or the chin, suck-and-puff control, voice control, or eye-tracking systems are among potential devices that could be employed to assist motor-impaired people. In high SCI lesions, and particularly those depending on artificial ventilation, the input devices for setup of an electronic user interface are in general very limited and may not work with a sufficient level of performance over an extended period of time. Computer technology allows people to access information on the Internet offers an alternative or additional means to communicate, and enables participation in education, work and leisure. Therefore, over the last decade Brain Computer Interfaces (BCIs) became an interesting option for end users who achieve only a moderate level of control with traditional input devices.

According to recent evidence the majority of SCI (68.6%) use computers and within this population, almost all of them use the internet and windows (11, 12). The paper of Rupp (3) addresses the factors limiting the use of BCI interface with SCI. In particular, persons with motor-related disabilities are often unable to use a standard keyboard. However, in cases where an individual exhibits motor and speech impairments, these solutions might be problematic because they lack efficiency for these populations. For example, scanning solutions are typically very slow although they require only a single input in the form of switch activation. Another limited option is speech recognition. Text entry using speech can be fast, but speech recognition requires the ability to enunciate clearly. Such a model is not an option for a user with NMD because of dysarthria. Questions therefore arise on how to support users with motor and speech impairments who are forced to invest considerable effort and time to work with a standard keyboard because other options are unacceptably slow or inaccessible? All these parameters should be taken into account when working with NMDs. In a survey with NMD patients regarding the desired BCI features priorities, it was found that 63% of the subjects currently use a computer while the rest have stopped (13, 14). No information was supplied regarding the preferred computer type or the operating system. According to Rimmerman (15) it is not just the physical immobility element that defines disability, but the socially constructed and propagated limitations that keep people with motor-impairment from having independent lives, and fulfilling relationships, roles and interactions. Access to the digital world is vital for being included and for having access to tools and information that heightens quality of life and quality of interactions, yet the manufacture of digital devices has not kept up with the specific needs of persons with disability (16, 17). Therefore, in the absence of specific, tailor made assistive devices, people with disabilities do not have access to digital spaces and are not offered equal chances for digital inclusions (18, 19).

Thus, the suggested solution is designed to be a medium that helps integrate people with disabilities back into society by offering them access to managing and authoring multimedia content, overcoming their inability or discomfort in using the digital world to connect with others and to be productive in social contexts. Our solution accomplishes this with the use of novel and more natural interface channels. It makes it possible for people with disabilities to control digital devices and to author content through the use of eye-movements and mental commands, thus increasing the ability to communicate and be socially included in important areas, such as work, education, social participation and entertainment. In particular our study has sought to fulfill and tackle the following elements: (a) it has moved away from using hand and voice in operating a digital device, to using eye movements and the mind, using electrical signals and bio measurements, creating a new paradigm of human—device interaction, (b) it has been tested and optimized over pilot trials using a sample of diverse potential users, whose immobility and physical symptoms are caused by diverse reasons, namely, SCI, PD, and NMD, (c) its efficacy and impact on social inclusion is measured following use over 1 month, employing social inclusion parameters.

### Social Inclusion as a Pivotal Pillar for Motor-Impaired People

A pertinent definition of social inclusion addresses not only social participation and an adequate share of available resources, but also participation in the determination of both individual and collective life chances (20). By contrast, social exclusion is the process in which individuals or entire communities of people are systematically blocked from (or denied full access to) various rights, opportunities and resources that are normally available to members of a different group and which are fundamental to social integration within that particular group (21–23). Social inclusion, the converse of social exclusion, is affirmative action to change the circumstances and habits that lead to (or have led to) social exclusion. Relevant to disability, the World Bank defines social inclusion as the process of improving the ability, opportunity, and dignity of people, who may be disadvantaged on any basis, to take part in society (24). And the digital landscape offers a very wide span of social interactions in a very compact form. As proposed by the social model of disability, it is not just the physical impairment that is relevant, but most importantly the consecutive socially constructed limitations in asserting an independent life and socially fulfilling roles and interactions (15). One such limitation is that of being excluded from digitally available resources and connections. A recent study defines digital exclusion as a phenomenon whereby marginalized individuals are not able to access and meaningfully participate in the same learning, employment, social, volunteer activities as others who have access to and use of digital devices like computers and smartphones (25). Moreover, the design and structure of digital devices has consistently neglected the specific needs of persons with disability (16, 17). This has meant that unless provided with specific assistive technologies, many persons with disabilities are significantly excluded from digital environments (18). At this point, extensive usability testing is performed on digital devices to ensure an easy, convenient, and accessible user experience. However, usability tests are not enough in considering the needs of persons with disabilities (26). As digital technologies and the world wide web continue to become more important in everyday life, equal access to digital devices will continue to grow in significance. Until the needs of persons with disability become a regular, ongoing consideration at the root of software and hardware development, our research approach development focuses on being a fully accessible, agile and efficient link with the digital space. Bowker and Tuffin (27) found the flexibility of online media provided control over people with disability's disclosure of impairment, an opportunity not typically available in real world social interactions. They could communicate through online media, without the element of their disability becoming disclosed, and without it becoming an element in interaction and communications. Based on a 1-year follow up questionnaire, their study indicated that those communicating with the help of ICT discovered a new sense of friendship and show significantly reduced isolation. People with disability find online self-help groups and blogging important for feelings of inclusion. McClimens and Gordon (28) conducted a study in which people with intellectual disability were introduced to, and trained in writing blogs over six meetings. The authors stated that the participants experienced a new form of inclusion and empowerment when able to express and share their thoughts and feelings online.

Access to information and services through websites, which in other contexts are hard to obtain or are unavailable, gives people with disability a sense of inclusion in society as a whole (29). Closely related to access to information and inclusion is a sense of empowerment. For people with disability, empowerment can be provided by the use of computers and the Internet (30), which facilitates them to make their own decisions. With the help of information and communication technology, people with motor impairment can have access to information needed to make decisions or acquire a sense of control over issues that concern them. Moreover, the digital and online landscapes may enrich the overall quality of life for people with disability and enhance their physical, emotional and social adjustment, through social interactions, employment and volunteer work opportunities (31). A previous study (32) notes the impact of online participation on psychological wellbeing. Participation in social networks is associated with psychological wellbeing (33, 34). Lee et al. (35) found a positive correlation between social network activity and subjective wellbeing in students with motor impairment. Thus, empowerment and subjective wellbeing are important indicators of digital inclusion. The purpose of the present study is to succeed in having a profound effect in how persons with motor disabilities become integrated in the society and have full and continuous access to all the resources and support systems they need, in order to have a fulfilling life and a rewarding sense of self identity. The appropriate indicators that can best evaluate the social effect of the proposed solution will have to be grounded on a foundation that defines disability with social rather than strictly medical and/or physical parameters. Indeed, a definition of disability that not only includes, but is based on the social isolation dimension would have far reaching implications. It means that disability is no more a product of the physical dysfunction, but of the social and emotional isolation that this dysfunction causes. Once this isolation is minimized, then the significance of the physical dysfunction in curbing options and opportunities also becomes minimized. The social model will, thus, be used here, to define disability and to serve as a compass in specifying the social inclusion indicators.

Our study's goal was to integrate motor-impaired people back into society by increasing their potential for social inclusion. In this direction, we delivered a technology platform, the “GazeTheWeb” (36) to enable interface channels that can be controlled through eye-movements and mental commands, by engaging three different cohorts of motor-impaired patients: SCI, PD, and NMD. On the other hand, our objective was to provide easy interaction capabilities to users with motor disabilities, so that they can operate various computer applications and accomplish their desired tasks. This was facilitated by the exploitation of novel sensors that can provide us with the necessary signals for natural interaction through the eyes and the mind. It is hypothesized that the use of “GazeTheWeb” will shift digital inclusion indicators: More time spent online, more extensive use of software like e mail clients, more active use of social media. It is hypothesized that a difference in the social indicator measurements will be evidenced, before and after the usage of “GazeTheWeb”. We also hypothesize that the social inclusion indicators may take a bit longer to show steeper changes. Though digital inclusion indicators may quickly show the difference impacted by “GazetheWeb”, however social indicators are based on deep seated habits and it is possible that these habits needs bit longer to transition. We hypothesize that our solution would have a positive effect on five digital indicator measures: active hours of usage, sites ever visited, keystrokes on the keyboard, clicks on screen and typing speed. Moreover, it was anticipated that the following measures would exhibit the impact of the proposed solution: number of sessions, total time spent and number of keystrokes in social sites. It is hypothesized that the participants will report satisfaction with using the suggested technology, and that they will express positive expectations influencing their sense of being included, as measured by the specific social indicators included in the questionnaire.

## Materials and Methods

**Figure 1:**
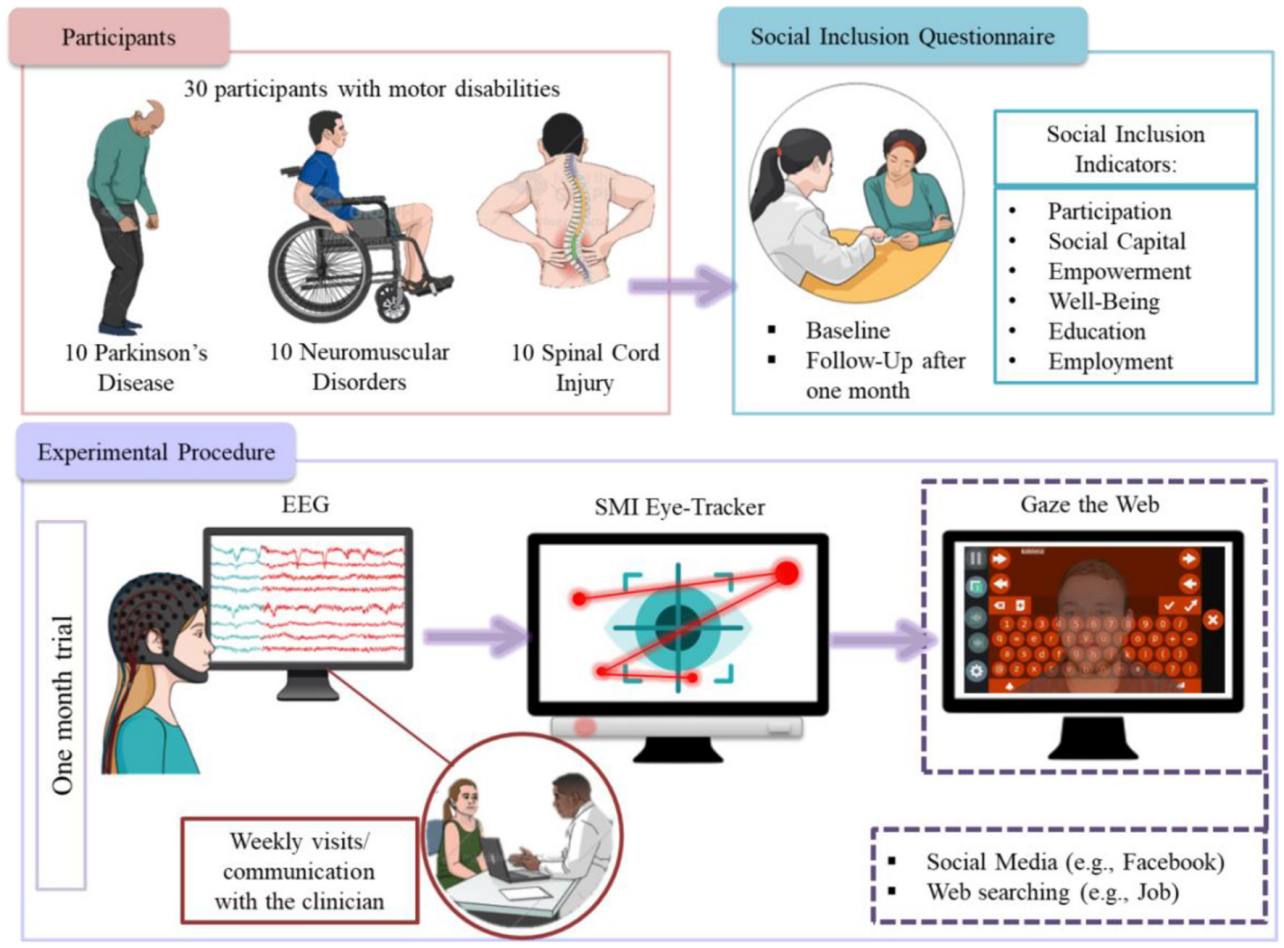
Pipeline methodology.

In the present study 30 motor-impaired participants used a platform in their home environment while implanted an EEG and SMI tracker (Figure 1). The platform was given to them for a fixed period of 1 month following a visit in which they were trained to use it. During this period the participants' activities performed in the computer were logged using the built-in monitoring mechanism of “GazeTheWeb” (36, 37), as well as using a tailored version of the Social Tracker that has been able to monitor their public online activities, as shared through their social network accounts. To allow privacy, the participants were able to turn off the “GazeTheWeb” logging mechanism while they also had the choice to keep their social activity among their friends network. On top of the monitoring software, data were also collected *via* participants responding to a series of questionnaire tools, namely, the social inclusion questionnaire, while they also responded in open questions related to social inclusion, during the use of the platform at home.

### Participants and Settings

A multi-center research design was used in the exploratory study involving volunteers living in different countries (Israel and Greece). From February 2018 to September 2018, participants were recruited from the 3rd Neurological Department of G.H. Papanikolaou of Aristotle University of Thessaloniki, Greece (http://www.med.auth.gr/), from the MDA-Hellas in Greece (https://mdahellas.gr/) and SHEBA hospital in Israel (https://www.sheba.co.il/). To answer our research hypothesis, a total of 30 individuals with disabilities were engaged 10 patients with SCI, 10 with PD, and 10 with NMD. The following areas were evaluated: (1) the impact of “GazetheWeb” on the use of digitally inclusive activities (e.g., use of Facebook), (2) the degree of satisfaction with the usage, (3) the perception of “GazeTheWeb” impact on feeling socially included given time and familiarization with the system after using it for 1 month. All participants were right-handed and fulfilled particular exclusion/inclusion criteria prior to their participation. In addition a pre-test was conducted in advance of apparatus home placement to ensure all participants were able to operate it, and thus minimize the possibility of dropping out. The pre—test consisted of a 5-min operation task and ensured that all participants would be able to handle the eye tracker apparatus. However, one SCI participant did eventually drop out of the study due to lack of motivation. Table 1 presents the demographic characteristics of the NMD, SCI, and PD participants.

**Table 1:** Demographic characteristics of the NMD, SCI, and PD participants (*n* = 30).

	**NMD**	**SCI**	**PD**
	**%/*M* (SD)**	**%/*M* (SD)**	**%/*M* (SD)**
**Age**	31.50 (4.8)	38.10 (10.77)	55.60 (7.32)
**Education**	15.50 (3.6)	13.10 (2.85)	16.20 (3.88)
**Gender (F:M)**	4:6	10:0	4:6
**Marital status**			
Married	20.0%	30.0%	100%
Single	80/0%	70.0%	0.00%
**Children no**.			
0	100%	70.0%	%0.00
1	0.00%	10.0%	30.0%
2	0.00%	10.0%	70.0%
3	0.00%	10.0%	%0.00
**Employment status**			
Full-time	20.0%	10.0%	60.0%
Part-time	30.0%	30.0%	40.0%
No	50.0%	60.0%	%0.00

*Values are presented either in % or mean (standard deviation)*.

### The Apparatus

The apparatus of the 1-month trial included a standard laptop computer with “GazeTheWeb” installed on patients' home (36, 37). The laptops were relatively new with i5 6th generation Intel processors, 4 GB RAM and 240 GB SSD hard drives. For the gaze behavior analysis, the apparatus also included the SMI eye tracking system. It also included an EEG device that was used in collaboration with the experimenter during his first visit to operate the multi-modal interfaces of error-aware gaze-based keyboard and a hands-free version of the Tetris game (MM-Tetris) (38). The platform included the “GazeTheWeb” on each computer, in addition to supporting software for the trials. This supporting software included the TeamViewer application, which was for remote technical support, if needed. In addition, the platform included a built-it monitoring mechanism that recorded every action that the user performed with the system. This monitoring mechanism had a temporary “turn-off” option for privacy reasons. Another monitoring mechanism was the social tracker application, which monitored the public activities of the participants in online social networks. It is important to note that the “GazeTheWeb” platform was equipped with software applications carrying persuasive design elements that were incorporated with the purpose to encourage its usage, and to combat possible resistance to its use due to pre-existing habits or to inhibitions toward innovation (39). The procedure of the 1-month trial unfolded over three stages is described in Table 2.

**Table 2:** Procedure of the 1-month study.

Pre-trial preparation and training	a) pre—screening regarding the ability of the participants to use the eye tracking apparatus b) signing of informed consent c) completion of the social inclusion questionnaire (see Supplementary Material) d) installation of the apparatus e) complete explanation of the “GaseTheWeb” and the installed mechanisms f) training session g) participants were reassured that they could access help at any time
Follow up communication	At the 2 week platform usage milestone, each of the participants was contacted *via* phone in order to check the progress of the trial, solve any issues and ensure smooth usage of the device
Post-trial visit	Following usage for 1 month, participants responded to the social inclusion questionnaire, and the system was removed from their home


**Figure 2:**
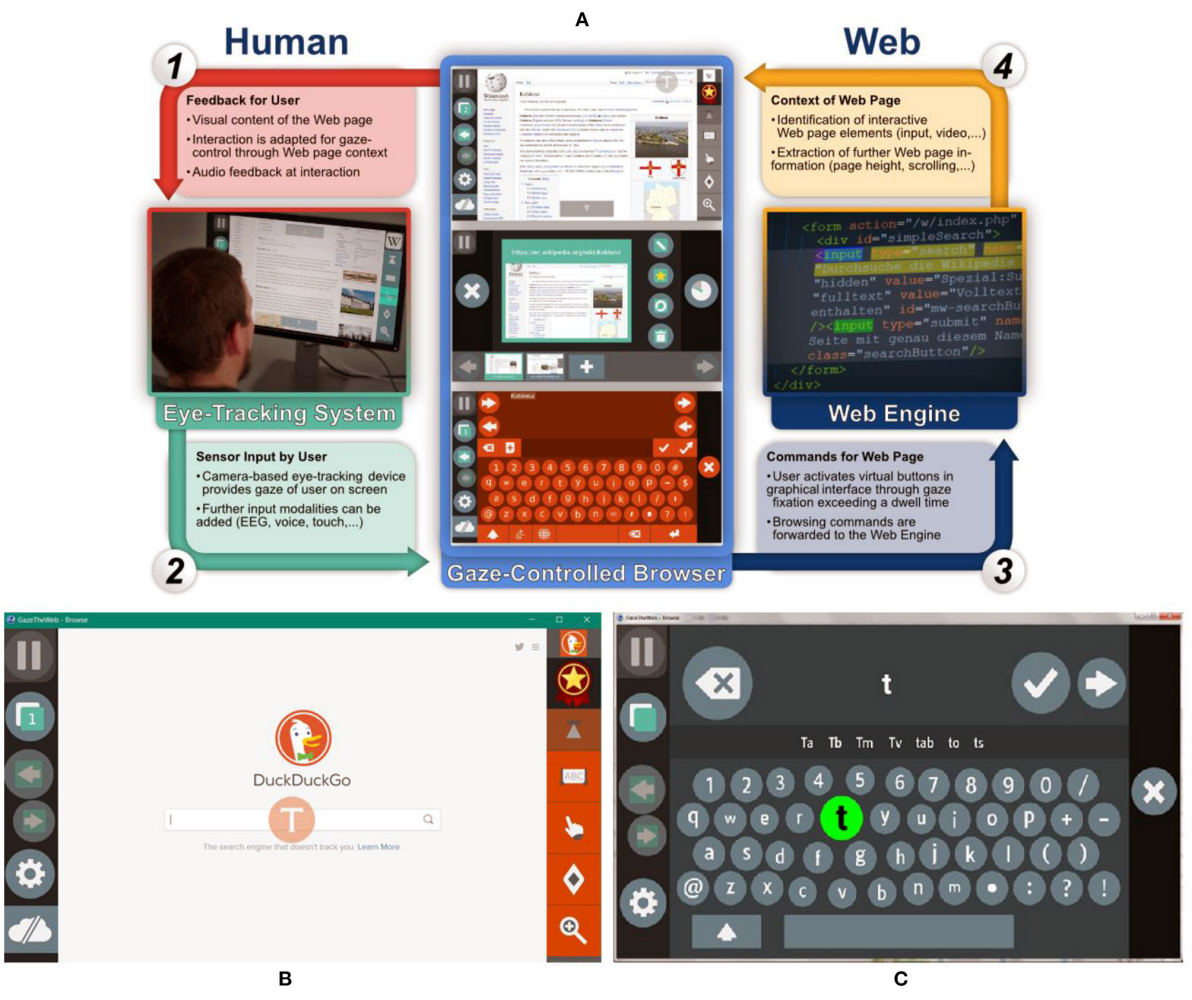
**(A)** The architecture of the “GazeTheWeb” browser, **(B)** Indicative example of the “GazeTheWeb” interactive interface, and **(C)** The gaze-based keyboard.

The intention for “GazeTheWeb” is to solve a core issue that people with disabilities have currently (even when they are willing and able to communicate digitally and to establish online relationships and networks), which is the ability to overcome the physical constraint of fatigue that results from having to use muscle operated devices. On the other hand, the eye-tracker provides a natural way to interact and assist physically disabled people to communicate with computers. For this reason, we chose the analysis and realization of eye tracking signals as the primary interaction mechanism between the user and the application platform. Our approach is to acquire a user's eye gaze information in real-time by an eye tracking device that can be used to generate gaze events, and to analyze the data to deduce more high-level events. In this direction, we present a novel browser, the “GazeTheWeb” that consists of a communication path between the eye tracker and the application interface, with the architecture of the proposed browser being graphically illustrated in Figure 2A. It allows the creation of interactive applications with many vital aspects, like rendering, layout, dynamic modification of content, support of graphics and animation. “GazeTheWeb” browser is a Web surfing system for adapted interface and functionality for eye tracking based input signals. More specifically, once the browser is activated the navigational and interactional inputs that are traditionally generated *via* the mouse and keyboard are overridden by eye movement commands. Additionally, every selectable and interactive object of a web page (e.g., text input fields, hyperlinks, scrollable sections, or select fields) is identified by the “GazeTheWeb” framework and is represented with explicit and implicit indicators to be accessed by eye gaze input. This allows major functionalities like selection, scrolling, link navigation, typing, etc. to be performed *via* eye commands. In addition to control paradigms, “GazeTheWeb” browser provides an overlay of self-explanatory buttons that goes beyond the conventional menu-based functionality to assist gaze-based interactions. Figures 2B,C illustrate indicative examples of the interface and the capabilities of the “GazeTheWeb” browser. In Figure 2B we can see how the interface is embedded within a traditional browser. More specifically the left and right panels of the figure showcase the gaze-based functionalities (e.g., the activation of the gear icon in the left panel transfers the user to the browser's setting and preferences tab), while an interactive text input field can be identified at the center of the Figure (i.e., the letter T enclosed in an orange circle). The activation of the previously identified text input field results in the activation of the gaze keyboard shown in Figure 2C, where users can type using only their eyes.

**Figure 3:**
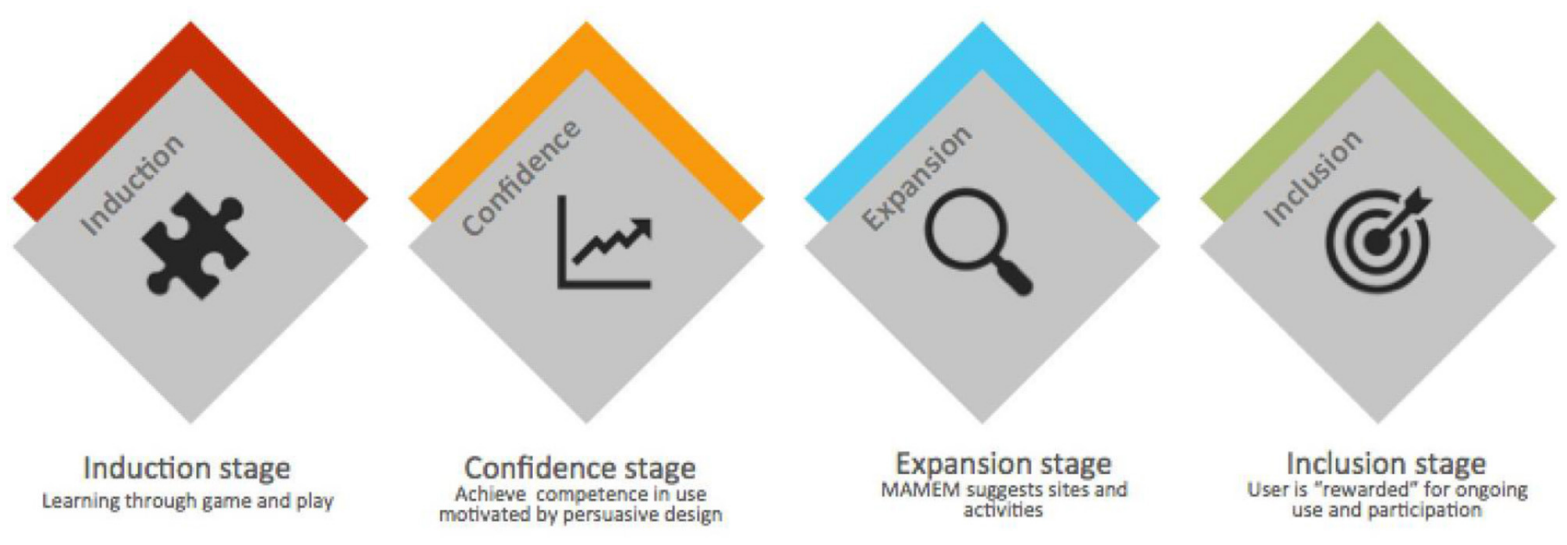
A four stage model to augment efficacy of “GazeTheWeb” in encouraging social inclusion.

“GazeTheWeb” will need to not only provide solutions for physical disability, but it will also need to tackle user mindsets. It will need to be an agent of attitude change as well, with regards to technology, and to what people believe they can do with technology. Given the findings of the clinical phase II trial, a four stage model is proposed, in order to build up “GazeTheWeb” efficacy in encouraging social inclusion (Figure 3).

The four stages are proposed as follows:

**Stage 1: Induction**

**Figure 4:**
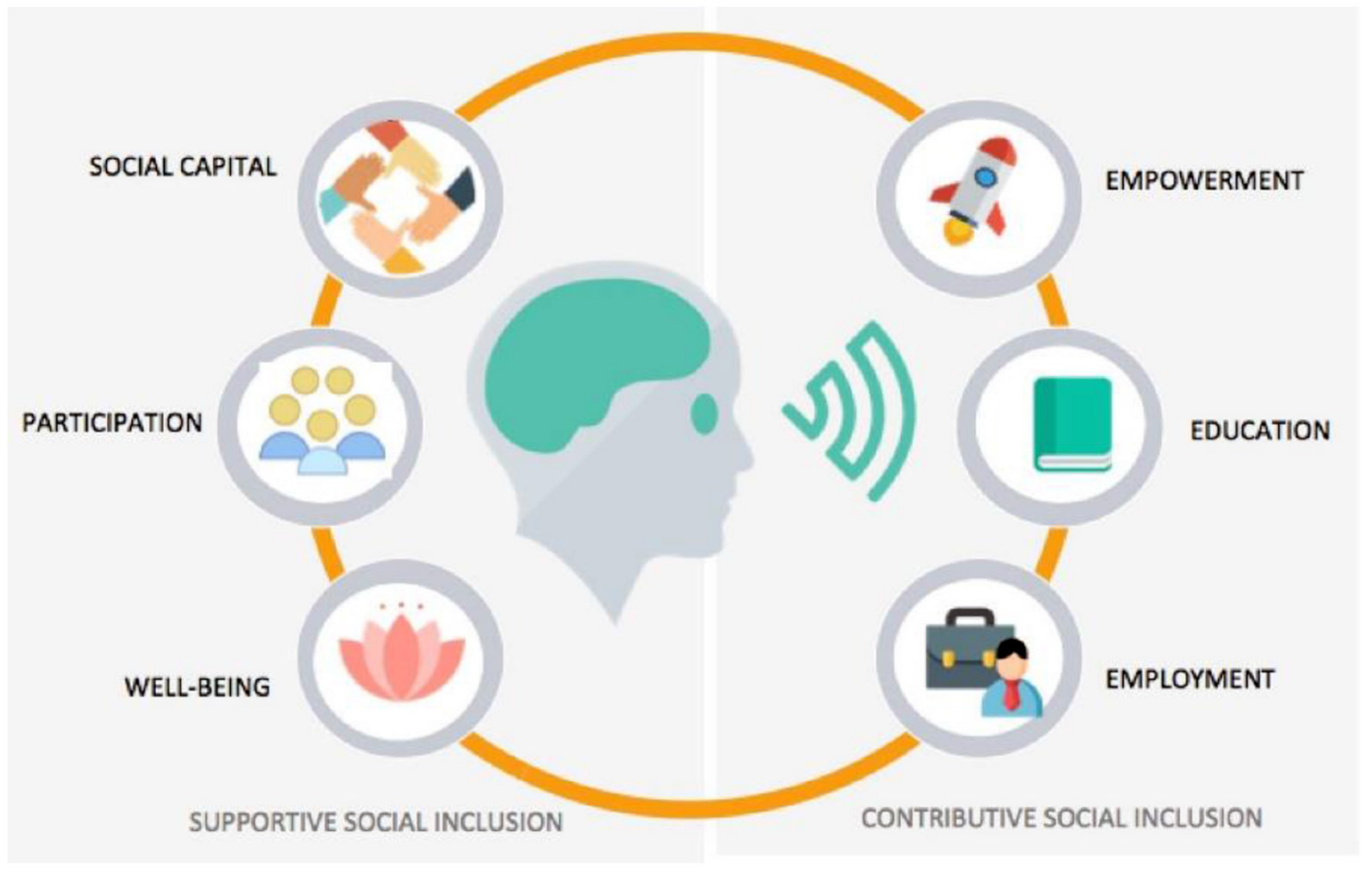
A “GazeTheWeb” model of supportive and contributive social inclusion indicators.

At this stage appropriate gaming is employed to make learning the system attractive and enjoyable. We need to make sure those vicarious learning videos and tutorials are available, which also integrate a sense of pride in being able to use this progressive technology (Figure 4).

**Stage 2: Confidence**

“GazeTheWeb” will be rewarding the user in a gamified way, reinforcing a sense of confidence about using the system, and fueling the motivation to keep using it.

**Stage 3: Expansion**

The system proposes tasks and activities relevant to the user, encouraging their expanded and more daring use of digital environments and resources.

**Stage 4: Inclusion**

The ultimate challenge for “GazeTheWeb” will be to form a user community and to turn its users into ambassadors offering a sense of pride in usage, as well as a sense of belonging and camaraderie with fellow users, in a “GazeTheWeb” community.

### Social Inclusion Indicators Questionnaire

**Figure 5:**
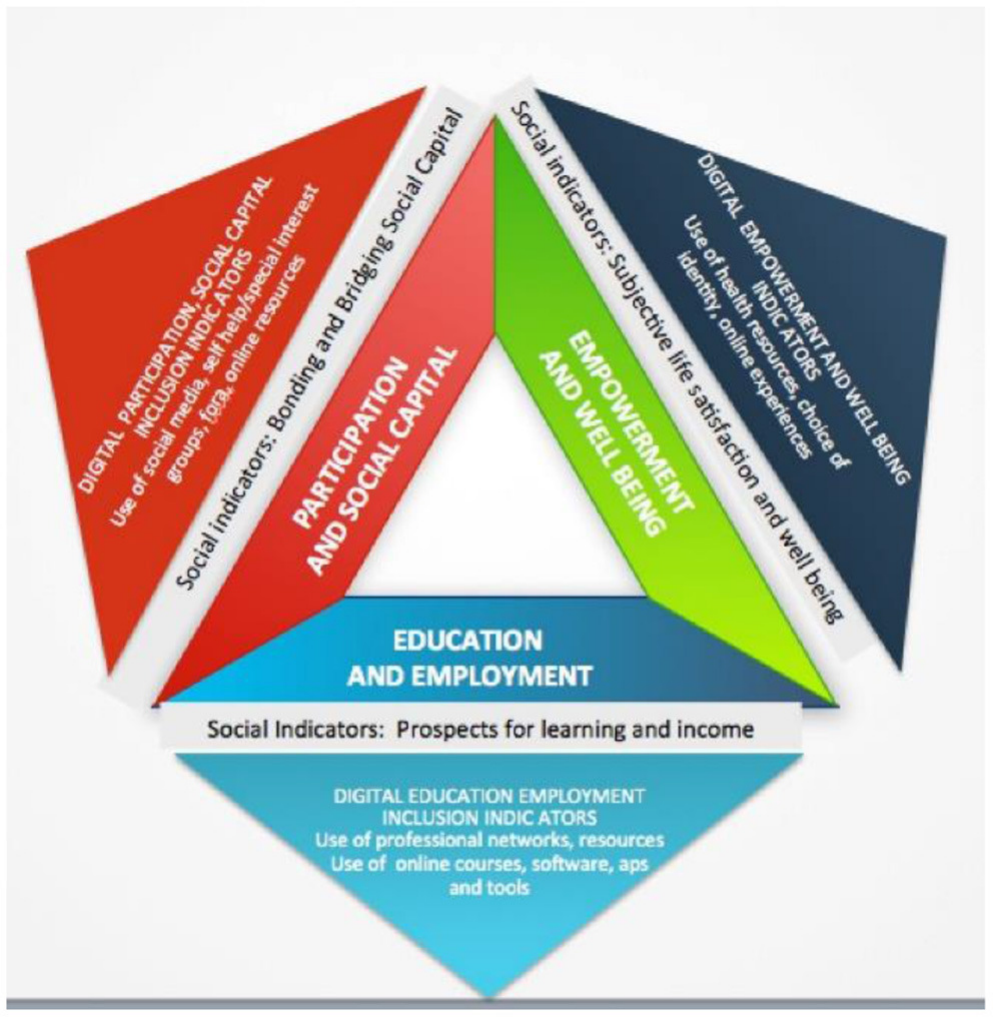
An integrated system of social inclusion indicators.

A portfolio of social and digital indicators is proposed that is founded on a triangle of three main axes: (a) The Education and Employment indicator axis, (b) The Participation and Social Capital axis, and (c) The Empowerment and Well Being axis. Each of these axes will have to be evaluated through social inclusion indicators (Figure 5). We have based our study on similar approaches that included similar open-ended and close-ended questions to assess end-users' requirements, views and concerns (40). In particular, we followed the responsive usability study design, which is most suitable to gain an understanding of the meaning of experiences (41–46), and focus mainly on the people's significant participation in all aspects of the research process (47, 48). Therefore, the pool or set of questions, referred to as a questionnaire in short, does not constitute a validated neuropsychiatric questionnaire, but rather one for eliciting requirements for interactive BCI “GazetheWeb”. The questionnaire includes three dimensions and was used in this study to explore the participants' requirements and preferences after using “GazetheWeb”. In this context, the three axes of social inclusion indicators will be used to measure social inclusion shifts, specific online activities that are hypothesized and outcomes experienced by the person with disability, following usage of the platform. Therefore, by measuring both social and digital inclusion indicators the research among SCI, PD and NMD individuals will verify the extent to which social inclusion outcomes are impacted by the change the platform brings to its users in facilitating their online activities. The indicators employed reflect the “5 Cs” of digital inclusion, which are: connectivity, content, continuity, confidence and capability. The Social Inclusion Indicators questionnaire used in the 1 month trial before and after participations incorporates elements from the research tools (Table 3) can be found in Supplementary Material.

**Table 3:** Research tools per social inclusion indicator.

**Indicator**	**Research tool**	**Validation of tool**
Bonding social capital	Internet social capital scales	(49)
Bridging social capital	Internet social capital scales Mail exchange SMS exchange Mobile access Social media account Frequency of interactions with friends online Participation in groups and fora Content generation	(49)
Personal empowerment	Shpigelman online experiences	(32)
Subjective well being	Warwick Edinburgh Mental Well Being Scale Entertainment (YouTube) Access to health related resources	(50)
Employment opportunity prospects	Social and community opportunities profile Business website owned Client and job search online	(51)
Education attainment prospects	Social and community opportunities profile Participation in e—learning Participation in professional social media	(51)

### Data Collection and Processing

Data was collected from the following sources in order to track and study the impact of the platform on social inclusion activities: (i) the monitoring apparatus which covered both computer usage monitoring as well as the tracking of activity in social sites, (ii) the social inclusion questionnaire administered before and after platform usage, and (iii) experimenter feedback. Due to the nature of the study, descriptive statistics were used to analyze the data of the social inclusion questionnaire. A *p*-value of 0.05 was used in all of the statistical analyses to assert statistical significance. Moreover, a qualitative analysis perspective was included in the study. Case study analysis is especially important if the questions that need to be tackled call not just for a description of phenomena, but also for their explanation. Through the case study analysis we will seek to enrich our understanding of the platform impact among the one-month trial. More specifically the multiple case embedded design of case study analysis was employed. According to Yin (52) each case must be carefully selected so that it either (a) predicts similar results or (b) predicts contrasting results but for anticipatable reasons. Thus, for each cohort a few participants were selected for a case study analysis, based on how frequently they used the system.

### Statistical Analysis

To obtain the statistical results the SPSS v25 and R studio program were used. The analysis was carried out per Social Indicator, tracking differences in responses to the social inclusion questionnaire used before and after the 1 month trial. Since there was no normal distribution among the data and due to the small sample size, non-parametric statistical testing (Wilcoxon Singed Rank Test) was performed, with a level of significance of 0.05. Moreover, in order to explore the potential correlation between clinical (e.g., severity of the disease) as well as demographic characteristics (e.g., education) of the participants and their responses in the “After” questionnaires, a Spearman rho Correlation Analysis was performed.

## Results

### Social Inclusion Indicator Questionnaire

#### The Participation Inclusion Indicator

In Table 4, the median response is charted for each question (Q1.1, Q3.5, Q3.6, Q4.6, and Q4.8—Supplementary Material), which evaluates the “Participation” Social Indicator, “Before” and “After” the one-month trial. Moreover, the *p*-value related to the statistical comparison of the questions is presented. It can be seen that although no statistically significant difference was found between the two evaluations in the majority of the questions, in Q3.6 an increase was found in the responses of NMD participants, who responded with 4.00 (partially true of me), where “before” the 1 month trial, the majority had responded with 3.00 (moderate/ mixed feelings). With regards to the SCI, it can be seen that there is an upward statistical significant difference in the responses to Q3.5, where the median response was 1.5 before the trial and escalates to a median 4.0 after the trial. Likewise, responses to Q3.6 have a tendency (though not statistically significant) to be higher after the trial. Regarding the PD, it can be seen that between the two evaluations, in Q1.1 after the trial the median response was 5.0 in a 5-point Likert scale, while it was 3.50 “Before” the trial.

**Table 4:** Descriptive statistics (median and standard deviation) for “Before” and “After” answers on participation social indicator questions and comparison using Wilcoxon non-parametric statistical test.

	**NMD**			**SCI**			**PD**		
**Using the platform over time**	**Before**	**After**	***P***	**Before**	**After**	***P***	**Before**	**After**	***P***
Q1.1 Given my disability I feel more included in life around me	4.00 (0.99)	4.00 (0.73)	0.32	4.00 (1.05)	3.00 (1.13)	0.10	3.50 (1.65)	5.00 (1.41)	0.52
Q3.5 My online interactions make me want to try new things	3.00 (1.10)	3.00 (0.63)	0.41	1.50 (1.28)	4.00 (1.62)	**0.03**	1.00 (1.75)	2.00 (1.58)	0.89
Q3.6 My online activities make me feel part of a larger community	3.00 (0.69)	4.00 (1.07)	1.00	3.00 (1.64)	4.00 (1.73)	1.00	2.70 (1.89)	3.00 (1.59)	0.67
Q4.6 I feel I can communicate and flirt with the other gender	4.00 (0.97)	4.00 (1.43)	0.86	4.00 (1.94)	4.00 (1.60)	0.32	1.00 (1.39)	1.00 (1.45)	1.00
Q4.8 I have the opportunity to volunteer and support others	4.00 (1.10)	4.00 (0.91)	0.65	4.00 (1.70)	4.00 (1.59)	1.00	3.00 (1.69)	3.00 (1.51)	0.13

*Bold value means p value significance level α = 0.05*.

More specifically, two NMD participants responded with “definitely true for me” in the Q3.6 question after the use of the system, which in terms of clinical characteristics the two NMD participants were those with the longest NMD diagnosis, that is, both have been diagnosed with NMD at the age of 1 year approximately. Even though the sample is extremely small, this common element of early disease onset points out to a hypothesis that the longer a person has suffered with debilitating symptoms, the more receptive they may be to platform's potential in impacting their lives, and are able to see its potential even within the very short duration of 1 month usage. Also, it is interesting to note the reaction of one PD participant, a 72-year-old male, a pensioner. Before the trial he responded with 1 (not true of me at all) to the question “I feel more included in life around me”. After the trial he responded with a 5 to this question, while during the assessment he stated “*After a very long time I was able to use the platform with no help at all from my wife, I was able to send an email completely on my own, without her help, and this has given me a lot of courage… it is like getting back my old self”*. With regards to question Q3.5, we need to keep in mind that the average age of the PD sample is 55 years of age. According to Prensky (53), these individuals are the “digital immigrants”, who did not grow up with digital tools but have had to adopt them later in life at varying degrees of competence. Their first reaction is to seek out conventional solutions to issues that are better and faster tackled with digital means. Thus, their median reaction of 1.0 (not at all true of me) to the question Q3.5 before the 1-month trial is to be expected. However, that reaction is raised from a median of 1.0 to a median of 2.0 after the trial, suggesting that the platform might be found helpful even for this population. Question Q4.6 which has to do with flirting with the other gender is not relevant to this sample, as all participants are married, thus the low median response of 1.0 in the 5 point Likert scale is to be expected.

#### The Social Capital Inclusion Indicator

In Table 5, the median responses for each question (Q2.2, Q2.5, Q3.1, Q3.2, and Q3.3) can be found, which evaluate the “Social Capital” Indicator, “Before” and “After” the 1 month trial. It was found that although there was no statistically significant difference between the two evaluations (“Before” and “After”), in Q3.1 and Q3.3 the responses followed an upward trend after 1 month usage for NMD. Additionally, more positive responses in the “After” evaluation in Q2.5 where the majority answered 4 and nobody answered 1 as happened in the “Before” evaluation. This indicates a fairly high receptivity of the participants to the platform impacting their social capital practices. The responses remain low to the question Q3.3. The low median response of 1 out of 5 to this question both before and after the trial indicates that loneliness is a deep and far reaching issue in the life of the SCI, which probably cannot be tackled singularly through digital social inclusion. Moreover, it is interesting to note that there is a negative trend in reactions of the PD (though not statistically significant), which however may be hypothesized to be an indication of the resistance to novelty that can be present in people of older age groups.

**Table 5:** Descriptive statistics (median and standard deviation) for “before” and “after” answers on participation social indicator questions (before and after) and comparison using Wilcoxon non-parametric statistical test.

	**NMD**			**SCI**			**PD**		
**Using platform over time**	**Before**	**After**	***P***	**Before**	**After**	***P***	**Before**	**After**	***P***
Q2.2 Contributes to my active participation in social sites	5.00 (0.97)	4.50 (0.69)	0.71	3.00 (1.39)	4.00 (1.48)	0.19	1.00 (1.37)	1.00 (1.50)	1.00
Q2.5 Contributes to my participating in groups and fora	3.50 (1.51)	4.00 (1.07)	0.76	1.00 (0.48)	2.00 (1.30)	0.18	1.00 (1.56)	1.00 (1.53)	0.59
Q3.1 I find there are people online to support and help me	3.00 (1.03)	4.00 (1.41)	0.34	2.00 (1.49)	3.00 (1.39)	0.46	4.00 (1.83)	3.00 (1.69)	0.83
Q3.2 I find people to turn to for advice	3.00 (1.43)	3.00 (1.17)	0.68	3.50 (1.41)	4.00 (1.58)	0.71	3.50 (1.91)	1.00 (1.54)	0.14
Q3.3 When I feel lonely there are people online to connect with	4.00 (1.43)	4.50 (1.33)	0.12	1.00 (1.49)	1.00 (1.41)	0.16	2.50 (1.93)	1.00 (1.66)	0.10

The hypothesis is that usage of social sites, groups and fora is not perceived to be relevant to this age group. The fact that median responses to the questions on social sites, groups and fora, both before and after the trial are so low is a clear indication of the digital exclusion of the older age groups. In general, research studies discuss the tradition barrier to innovation in older people, describing it as resistance to changes in daily routines and habits (54), while others present the notion of passive innovation resistance, which is related, again, to satisfaction with the status quo in older people (51). The slow reaction of more mature people, like PD, to innovation like the suggested platform is expressive of their propensity to digital exclusion. Albeit, in terms of clinical characteristics an NMD with severe paralysis in the majority of upper limbs and partial paralysis in his tongue, answered 1, before the platform installation, while the participation in 1-month trial the participant responded with a 4. This underlines the contribution of the system toward patient engagement in groups and fora, which is very important in terms of social capital. We see that 3 out of 10 NMD participants responded with “fairly true of me” in the Q3.1 after the use of the platform, who had the most severe immobility and paralysis in the upper limbs. This finding suggests that even after the short duration of the trial (~30 days) these three NMD participants with the most severe motor impairment had a more positive reaction to using the platform. In addition, we found an upward trend in responses with regards to Q3.3, where 5 out of 10 NMD participants responded with a 5 and two with a 4. Both findings drive the way to suggest that the usage of platform helped the participants to address the feeling of loneliness and support. According to research loneliness is one of the most debilitating characteristics experienced by people with disabilities; therefore the positive reactions described above can be readily understood (51). We propose the platform as an easy and user friendly tool that can be an answer to this exclusion, should it be used in the longer term.

#### The Empowerment Inclusion Indicator

A statistically significant difference in Q1.3 was found with regards to the empowerment inclusion indicators (Table 6), where the participants answered with a 5. With regards to the questions Q1.4, Q3.7, and Q3.8, although there was no statistical significant difference between the two evaluations (“Before” and “After”), in Q1.4 there was an upward trend in the responses of NMD participants. In particular, 5 of 10 NMD responded with a 5, whilst in the “Before” evaluation there was greater variability in responses. More specifically, an NMD patient answered 1 in Q1.4 in the “Before” installation questions, while in the “After” questionnaire she answered 4. Both findings drive the way to suggest that the use of the platform helped the participants to address the feeling of personal esteem and usefulness. The indicator of “Empowerment” is strongly related to the sense of accomplishment and personal value. It can be hypothesized that getting in touch and using state of the art, progressive technology, does provide people with disability with that sense of accomplishment and self-value. In SCI population, though there are no statistically significant differences in responses “Before” and “After” the trial, it can be seen that there is an upward trend in the responses to questions Q3.7 and Q3.8 which went from a median of 2.50–4.00 out of 5.0. The response to the question Q1.3 was high both before and after the trial. This may well mean that the SCI participants in the sample, who suffer from debilitating immobility symptoms, are already fully aware of the significance of digital devices. This finding underlines the contribution of the system toward using specialized software for hobbies and entertainment, which is very important for the psychological uplifting of people with disabilities from such an early age.

**Table 6:** Descriptive statistics (median and standard deviation) for answers to empowerment indicator questions and comparison using Wilcoxon non-parametric statistical test.

**Using the platform over time**	**Before**	**After**	***P***	**Before**	**After**	***P***	**Before**	**After**	***P***
Q1.3 Contributes to feeling I am playing a useful part in society	4.00 (1.10)	5.00 (0.96)	**0.05**	4.00 (1.43)	4.00 (1.00)	0.48	4.50 (1.48)	5.00 (1.64)	0.86
Q1.4 Contributes to my feeling valued by others	4.00 (1.05)	4.50 (0.82)	0.45	5.00 (1.05)	4.00 (0.83)	0.16	5.00 (1.37)	4.50 (4.67)	0.49
Q3.7 Contributes to my being active and creative in online activities	4.00 (0.88)	4.00 (0.74)	0.48	3.00 (1.59)	4.00 (1.58)	0.74	2.00 (1.89)	4.00 (1.74)	0.19
Q3.8 My online activities give me a sense of freedom/choice	4.00 (0.88)	4.00 (0.92)	0.78	2.50 (1.70)	4.00 (1.59)	0.29	2.50 (1.70)	4.00 (1.61)	0.48
Q2.1. Contributes to my active use of digital technologies	5.00 (0.70)	5.00 (0.71)	0.74	4.00 (1.65)	4.00 (1.27)	0.71	4.00 (1.40)	4.00 (0.83)	0.71
Q2.9 Contributes to my using specialized software for hobbies	3.50 (1.43)	4.50 (1.11)	0.14	2.00 (1.39)	3.00 (1.41)	0.49	1.00 (1.63)	1.00 (2.00)	0.46

*Bold value means p value significance level α = 0.05*.

As already mentioned, the SCI participants in their totality are fairly digitally savvy, and the platform contributes incrementally to their digital daily experiences. This can be seen in Table 6 clearly, noting the high median responses both before and after the trial to questions on digital empowerment indicators like Q2.1. Overall, there are no statistically significant differences in reactions before and after the trials, but there is an upward trend in responses, in Q2.9. With regards to the digital Empowerment indicators in PD group there is no statistical significance in answers “Before” and “After” the trial in questions relating to the use of digital technologies and the specialized software for hobbies. This, again, can be understood by the type of relationship this age group has with digital technologies. Van Dijk (55) describes that the physical digital divide across age groups may be closing, but the divide in digital skills and use persists. The studies of Li and Ranieri (56) and Salajan et al. (57) show that age is significantly and inversely related to digital fluency. The presenting results in Table 6 are in line with these studies, as relating to PD participants with a median age of 55 years.

#### The Well Being Inclusion Indicator

In this Section, the potential benefits of the system as regards to the Social Capital Inclusion Indicator are explored. More specifically, in Table 7, we can find the median response for each question (Q1.2, Q2.11, Q3.3, Q4.5, and Q4.7), which evaluates the “Well-Being” Social Indicator, “Before,” and “After” the 1-month trial. It was found that although there was no statistically significant difference between two evaluations (“Before” and “After”), an increase of the responses of NMD participants in Q3.3 and Q4.5 was encountered. More specifically, 5 out of 10 NMD participants responded with 5.00 in the Q3.3 question after the use of the platform. This finding suggests that even after the short duration of the trial (~ 30 days) these five NMD participants with a most severe motor impairment experienced a higher sense of wellbeing by using the platform. In addition, there was found a noteworthy increase in the median of responses with regards to Q4.5, where 6 out of 10 NMD participants responded with a 5 and 3 with a 4. Both findings drive the way to suggest that the usage of the platform helped the participants to address their need for fun and entertainment, as well as their need to access health information and support.

**Table 7:** Descriptive statistics (median and standard deviation) for answers to the well being indicator questions and comparison using Wilcoxon non-parametric statistical test.

**Using “GazeTheWeb” over time**	**Before**	**After**	***P***	**Before**	**After**	***P***	**Before**	**After**	***P***
Q1.2 Contributes to my feeling more optimistic	4.00 (0.88)	4.00 (0.63)	0.26	3.50 (1.43)	4.00 (1.16)	0.32	5.00 (1.66)	4.00 (1.42)	0.71
Q2.11 Contributes to my finding support and hiring help	4.50 (1.29)	4.50 (0.92)	0.62	1.00 (1.70)	3.00 (1.25)	1.00	2.50 (1.81)	1.00 (1.72)	0.46
Q3.3 I engage in online activities that entertain me	4.00 (1.43)	4.50 (1.33)	0.68	1.00 (1.49)	1.00 (1.41)	0.16	2.50 (1.93)	1.00 (1.66)	0.10
Q4.5 I feel I can learn more about health issues	3.00 (0.63)	5.00 (0.71)	0.09	4.00 (1.19)	4.00 (1.05)	0.41	4.00 (1.71)	3.00 (1.24)	0.32
Q4.7 I have opportunities to advance my hobbies and creativity	4.50 (0.82)	4.00 (0.74)	0.32	4.00 (1.52)	4.00 (1.50)	1.00	3.50 (1.57)	3.00 (1.48)	0.74

The SCI participants give high responses to the Well Being questions, both before and after the trial. For example they give a median response of 4.0 to the Q1.2, Q4.5, and Q4.7 questions. This finding is well aligned with the fact that all of the SCI respondents are heavy users of smartphones, keep them always by their side, and use them for long stretches of time on a daily basis. These participants suffer from severe mobility issues and the mobile phone can be their lifeline through the day. As one participant puts it, “I always keep my smart phone next to me, I even sleep with it, and I feel insecure without it”. Therefore, it can be concluded that for these participants the sense of Well Being is already associated with digital connection *via* the mobile phone. However, there is an upward trend from a median 1.0 to a median 3.0 out of 5.0, in the response to Q2.11, showing that the platform make a dramatic difference both in the quality of their digital connectivity, as well in other spheres of social inclusion.

With regards the PD group, there was a downward trend, though not a statistically significant one. One participant responded with a 5 to the Q3.3 question before the trial, and with a 1 to the same question after the trial. The 72-year-old male participant, who uses the computer mostly to browse on his military interests. He expressed delight and excitement about the platform at the first day of the trial, when the first interview took place. However, as the trial progressed he expressed that “I am struggling getting used to platform, I am a simple user of the computer, and I guess it might be easier to stick to my old machine…”. The participant can be said to be fairly typical of the digital exclusion that is characteristic of people in older age groups. They experience awe toward new technology, they struggle in the adoption process, and react by retracting from its use. This is all the more reason to create systems that will make it enticing for such individuals to be part of the digital world and its resources. The fact that the median response to the Q1.2 question was a median 4 after the trial indicates that the platform is such a technology that can captivate emotions. Its use over longer stretches of time could, and with further work on the integrated persuasive design would be an efficient vehicle for digital inclusion. Moreover, the question Q3.3 was statistically positively correlated with the number of years the PD participant has suffered from the disorder, suggesting that the longer the person has suffered from PD the more appreciative they grow of the chance to engage in online activities that entertain.

#### The Education Inclusion Indicator

Moreover, the potential benefits of the system as regards to the Education Inclusion Indicator were explored. More specifically, in Table 8, we have added the median response to the questions Q2.3, Q2.4, Q2.8, and Q4.2 which evaluate the Education digital social indicator, “Before” and “After” the one-month trial. More specifically, although there were not any statistically significant differences between “Before” and “After” responses as regards the Education, we found in Q2.3, Q2.4, Q2.8, and Q4.2 an upward trend in the responses of NMD participants. With regards to the PD participants, acquiring new skills gets answers with a high median both before and after the trial, indicating again that these participants are digitally rather savvy and we hypothesize that their use of mobile smartphones caters to these needs to a good extent. Moreover, this can be understood again in light of what is typical among older populations. Culturally, learning new skills is not part of their priorities. We consider this to be evidence of the educational exclusion prevalent in the culture, and we propose that the platform can be an answer to that exclusion, on the premise that it is accompanied with persuasive elements, which need to be further researched, which cater to impact the mindset of older sufferers about education. On the other hand, for SCI, it can be seen that there is an upward trend in the responses to questions Q2.3 that goes from a 1.0 median to a 3.50 median. Therefore, SCI participants seem to be interested in knowledge but not in education *per se*, as Q2.4 after the trial goes from a median of 1.0 to only 2.0, respectively. With regards to watching and reading content and videos the response is fairly high both before and after the trial, indicating that participants are already feeling fairly sufficient in this regard, possibly through their mobile smart phones. The responses in Q2.3 and Q2.4 reflect a pattern typical of cultural stereotypes for this age group. When it comes to using the Internet and content videos in the familiar way of watching TV, the responses are high (5 on a 5 point Likert scale). When it comes to using the Internet for education participants react in stereotypical ways, given that 4 out of 10 are pensioners, and the rest of them are near pension age. Their responses reflect a lack of interest in promoting their education, because both before and after the trial the responses to the education questions are low. We propose that this is an indication of digital exclusion related to a cultural stereotype whereby the need for education goes down as age advances. The suggested platform is an answer to this digital exclusion, with the condition that it is used long enough to counteract these stereotypes and to contribute to these people forming new attitudes to education.

**Table 8:** Descriptive statistics (median and standard deviation) for answers to the education digital indicator questions and comparison using Wilcoxon non-parametric statistical test.

**Using the platform over time**	**Before**	**After**	***P***	**Before**	**After**	***P***	**Before**	**After**	***P***
Q2.3 Contributes to my active participation in business, education sites	3.00 (1.56)	4.00 (0.99)	0.15	1.00 (1.46)	3.50 (1.47)	0.10	2.50 (1.51)	1.00 (1.76)	0.68
Q2.4 Contributes to my attending online courses	3.50 (1.07)	4.00 (1.25)	0.71	1.00 (1.16)	2.00 (1.03)	0.58	1.00 (1.25)	1.00 (1.54)	0.75
Q2.8 Contributes to my watching and reading content videos	4.50 (0.69)	5.00 (0.85)	0.56	4.00 (1.23)	4.00 (1.05)	0.71	4.00 (1.23)	5.00 (1.17)	0.56
Q4.2 I feel I have access to opportunities to acquire new skills	4.00 (0.74)	4.00 (0.79)	0.65	4.00 (1.66)	4.00 (1.51)	0.56	2.00 (1.58)	3.00 (1.23)	0.26

#### The Employment Inclusion Indicator

In the following Table 9, we have added the median response for each question (Q4.1, Q4.3, and Q4.4), which evaluates the “Employment” Social Indicator, “Before” and “After” the 1 month trial. No statistically significant difference was found between the two evaluations. More specifically, in both evaluations there was a similar response in each question. Several elements, irrelevant to the use of the platform, may be behind the absence of a “Before” and “After” usage shift in responses to Q4.1, Q4.3, and Q4.4. For example, it is possible that the existing recessionary environment in Greece, the participants' country of residence, and the negative outlook of the Greek economy may be influencing the reactions to perceived opportunities to find employment, to create new business ideas and to pursue promising business contacts. In contrast, there were more positive responses to the platform in relation to the digital inclusion indicators, referring to having access to digital tools for employment. Employment is an important issue for the SCI participant, given the fact that their majority has had their injury at a very productive stage of life. However, the platform did not generate a significant shift in the employment related indicator questions. We propose that employment is a multidimensional issue in the case of SCI victims. Several of them either has very efficient support systems and dedicated care takers, or are receiving significant compensation for their injury. Thus, there is no motivation for employment. We propose that our definition for employment needs to be reworked to fit the specific needs of SCI victims. Employment need not be defined strictly in monetary terms (working in order to earn) but in terms of productivity, creativity and personal expression. Employment can be pursued not strictly for financial survival, but primarily for psychological and mental wellness, and sense of personal value. Moreover, the fact that employment is not a key issue in the specific life stage that PD sufferers find them in is expressed in, addressing the need for business ideas, business contacts and employment the PD participants respond with low scores overall. Four out of ten participants are pensioners and another six of them are near pension age.

**Table 9:** Descriptive statistics (median and standard deviation) for answers to the employment indicator questions and comparison using Wilcoxon Non-parametric statistical test.

	**NMD**			**SCI**			**PD**		
**Using the platform over time**	**Before**	**After**	***P***	**Before**	**After**	***P***	**Before**	**After**	***P***
Q4.1 I have access to opportunities to find employment	3.50 (1.35)	3.50 (1.26)	0.73	2.50 (1.52)	3.50 (1.64)	0.71	1.00 (1.35)	1.00 (1.13)	0.71
Q4.3 I have access to opportunities to create new business ideas	3.50 (1.35)	3.50 (1.32)	0.67	3.00 (1.70)	3.00 (1.36)	0.41	1.00 (1.52)	1.00 (1.52)	0.41
Q4.4 I can pursue promising business contacts	3.00 (1.25)	3.00 (1.45)	0.52	3.00 (1.56)	3.50 (1.64)	0.32	1.00 (1.32)	1.00 (1.32)	0.41

## Case Studies

In this section case studies of some individuals from each group will be analyzed, to gain more depth in understanding reactions to the platform usage (Box 1). Following the user-centered design (UCD), the case study analysis has the purpose of enriching our comprehension of the use of the platform by the sample of participants. It aims to aid the development of explanations of the behavioral, computer use and digital indicator data, as well as the reactions to the social inclusion questionnaire.

Box 1 **NMD 1 participant case study**

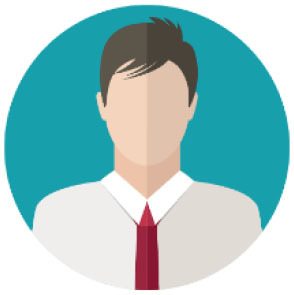

NMD 1 participant is a 36 year-old male, married, having no children. He was diagnosed with Muscular Dystrophy at the age of 6 and he has been living with this clinical condition for the last 30 years. Due to the disorder, he has severe paralysis of the upper limbs, and limited movement of the hands and fingers, which allow some use of the PC. For him to be able to be part of society, and live a normal life is of high importance. This person is fairly isolated at home, with very limited access to social activities out of home. Currently he also lacks a rich supportive social system, besides his immediate family. The use of the computer is his favorite, and often his only pastime, as he rarely ventures out of home, or receives visits at home. Nevertheless, he describes his social life as close to normal, and he has few complaints. He tends to be a fairly positive person, who takes his life conditions in stride. He is a clear example of the “reconstructed normalcy” that has been described earlier.

His platform usage

NMD 1 participant was very receptive to the platform and very eager to try on the device. He requested that all of the software he uses in his current device be moved to the platform laptop, so that he might completely substitute the computer used up to that time. From the data obtained while the NMD participant was online through the platform, it was found that NMD 1 had significant participation in social media accounts and he was regularly using educational sites. More specifically, MDA1 reached a high score on almost all Social Indicator parameters right from the start and improved his score till the end of the trial. Regarding the Social Indicator of “Participation and Social Capital” (E-mail, Social media, Fora etc.), we found that NMD1 reached the highest score of 83.48% during the trial, while the worse score in this indicator was 26.46%. As regards to the “Empowerment and Wellbeing” Indicator (YouTube, News, Entertainment, Health etc.) NMD1 participant reached the score of 59.74%. Regarding the “Education and Employment” Indicator (E-learning, pro networks, job search), he reached the score of 66.67%.

Success factors

Home confinement with long hours of boredom was a motivating element for this individual. In addition, the need to be self-sufficient was also a motivator. He saw the platform as a challenge where he had to prove himself, and he did so successfully. His lack of a strong social supporting system around him also energized him to tackle ‘GazeTheWeb' with enthusiasm to see what he could accomplish with it.

The implication

It is interesting to note that the long-standing social isolation has made this individual socially self-sufficient at much lower levels of social interaction. The poor levels of social interaction seem to have gone on long enough for the individual to have developed “a reconstructed normalcy”, where he feels good regardless of the poverty of his social integration. Thus, even though at the end of the trial NMD1 ended up being a power user of the platform, he stressed that he would substitute his current computer with the platform only should he loose complete mobility of his fingers. He prefers the use of his fingers to the use of his eyes even though the latter would give him an easier and speedier use of the digital environment. This individual has reached a point where for reasons of self-preservations, he no more “misses” the social inclusion he lacks. He does not experience a sense of poverty in social interactions. It is hypothesized that it will take longer use of a new device to reach a point where the need for social interaction is awakened and becomes ongoing again.

**NMD 2 participant Case Study**

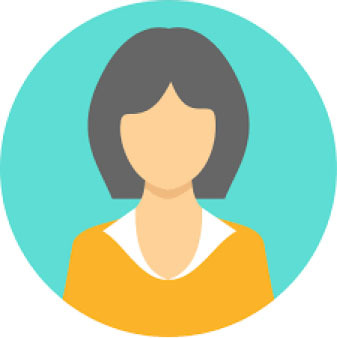

NMD2 is a 36 years old single woman with a degree in Philosophy who has a full time job as a special education school teacher. She has lost all mobility of arms and hands, and as a result is not able to use the computer at all. She is confined to the wheel chair at all times. Nevertheless, with a lot of family support she is able to hold her teaching job, at which she thrives and commands recognition and respect among colleagues and students. She has an active social life, mainly because her family focuses on making it possible for her to go out of home and participate in activities she wants. She is using an e-book reader, with timed page turning, and up to the point of the trial, this has been the only digital device she has had access to. Even though she is active socially, she still feels restricted in the opportunities she has for further growth, training, skill building and development.

Her Platform usage

From the data obtained while the trial for the NMD2 participant was online, we found that the NMD2 has an outstanding participation in social media accounts and she was regularly using educational sites. The system seemed to work and fit perfect in NMD's needs active participation. More specifically, NMD2 reached a high score almost in Social Indicator parameters from the initial stage and improved her score till the end of the trial. More specifically, we found that the NMD2 had an active participation during the whole duration of the trial (9/3/2018–6/4/2018). Regarding the Social Indicator of “Participation and Social Capital” (E-mail, Social Media, Fora etc.), we found that she reached the highest score of 62.20% during the trial, while the worst score in this indicator was 17.86%. As regards to the “Empowerment and Wellbeing” Indicator (YouTube, News, Entertainment, Health etc.), the participant reached the score of 50%. Regarding the “Education and Employment” Indicator (E-learning, pro networks, job search), she reached the score of 25%. This proves that in all Social Indicators, NMD2 had an active participation during the trial.

Success factors

This participant reported a very positive experience because it created a dramatic difference in her digital interactions. She had gradually lost ability to use a computer as her hands became progressively immobile. So with the use of the platform she went from zero use of a digital environment to a full use of a digital environment. This user is a star example of the mission and scope of the platform in offering social inclusion. “*To be able to turn the pages myself, when I read, gives me a sense of control that I had lost… also, the fact that I re-activated my Facebook account and found a few of my friends online, again, that was an awesome thing for me.”*

The implications

What is very important to note in the case of the participant is that the platform offered a critical improvement in her relationship with digital devices. Though NMD2 was also a heavy and happy user, his digital experience did not become as augmented through the platform, as was the case with NMD2. She went from almost zero usage of the computer, back to reclaiming usage similar to the time when her hand movements were not restricted. She re-opened her Facebook account, and wrote emails herself, rather than dictating them. This radical difference in her social activities, using the technology made her truly sad to part with the apparatus, and eager to use it when available to the public. In this case there was an immediate, discernible and tangible big difference in the range of activities made possible with the platform. In this case, there was complete inability to use the computer before the platform.

**SCI 1 participant case study**

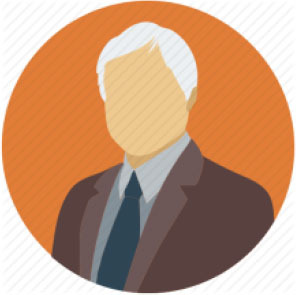

SCI 1 is a 60 years old married man with Spinal Cord Injury, which is located at C3. He has been diagnosed with non-traumatic SCI 4 years ago and since then he is using a motorized wheelchair. Although he is driving his own car and tries to be part of the society, his clinical condition forces him to be confined in bed for over 10 h every day, which causes him feelings of hopelessness and isolation. He tried out every possible solution that might enable him to return to his earlier condition. He has been attending a rehabilitation program for over 8 years, but without significant positive outcomes. Now, he suffers from complete paralysis of the hands and fingers and from partial paralysis of the upper limbs. He has accepted his clinical condition and he is more than happy to use technology in order to feel part of society and communicate with his daughter.

His platform usage

From the data obtained while the trial for the participant, we found that the SCI 1 has an outstanding participation in social media accounts and he was regularly using empowerment sites. The system seemed to work and fit perfect in SCI 1's needs active participation. More specifically, SCI 1 reached a high score in several Social Indicator parameters from the initial stage and improved his score till the end of the trial. More specifically, we found that the SCI 1 had an active participation during the whole duration of the trial (26/3/2018–24/4/2018). Regarding the Social Indicator of “Participation and Social Capital” (E-mail, Social media, Fora etc.), we found that the SCI 1 reached the highest score of 45% during the 1 month trial, while the worse score in this indicator was 6.25%. As regards to the “Empowerment and Wellbeing” Indicator (YouTube, News, Entertainment, Health etc.), the participant reached the score of 50%. Regarding the “Education and Employment” Indicator (E-learning, pro networks, job search), she reached the score of 20.7%. This proves that in all Social Indicators, the participant had an active participation during the trial. Regarding the “Education” as Social Indicator, given that SCI 1 is a pensioner and he does not like to visit educational sites, he did not use the system for such purposes.

Success factors

SCI 1 participant is an excellent example of successful platform usage for social inclusion. This participant is confined at home and rarely ever ventures out. Some of his favorite activities were to carry out online shopping and e- banking, and mentioned that the platform made these activities more enjoyable for him. Being able to shop online helped this participant reclaim a sense of control over his circumstances. He is virtually carried outside of home confinement into the virtual malls of the world wide web, being allowed to browse, shop around, and make personal choices again, rather than have others do the shopping for him. His ability to manage his own financial affairs, to carry out payments and track finances gave him a sense of empowerment, despite his confinement. In addition, this participant sought access to health resources actively.

Implications

In the case of SCI sufferers their physical vulnerability and confinement must be taken into consideration. Given the fact that like SCI 1 most SCI victims get easily tired and can use the platform for relatively short stretches of time and only in a sitting position, it will be important to develop solutions that will allow the use of the platform also in the reclining position, which is more comfortable to SCI patients.

**PD 1 participant case study**

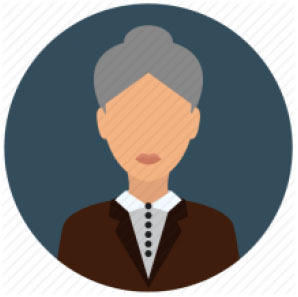

PD 1 is a 63 year's old married retired woman with two children. PD 1 has been diagnosed at the age of 46 with Parkinson's Disease and has partial tremor and immobility in the majority of upper limbs parts. Nevertheless, with a lot of her husband and children's support she is able to do the majority of households and she loves cooking. She has a very positive support system in that her family provides fully for her social needs. Her daughter is a physical therapist and provides almost daily physical care for her symptoms. The family bonds are very tight and she finds a lot of fulfillment in the contact and communication that she has with her grandchildren. For her, and for her life stage and culture, her perceived social needs are family related.

Her platform usage

She became enthusiastic about the use of the platform. Even though she did not use it heavily on a daily basis, the usage that she did was a very rewarding experience for her. She mentioned that she was able to use it beyond the gaming, for which the tablet fulfills her needs. She was able to search for cooking recipes, to exchange cooking recipes, to watch cooking videos and movies on YouTube, and to join groups with cooking as a hobby. It felt rewarding for her to be able to pursue her interest in cooking online, and to connect with others with a similar interest. She was also able to search for information and updates on her disease and symptoms. What is important to note is that the recommended sites, which were part of the Persuasive Design integrated into the system were a source of helpful suggestions for her, while also her children, knowing her interests, were able to recommend ways in which the platform could be interesting to her.

From the data obtained through “GazeTheWeb” Dashboard/Homepage (“GazeTheWeb” Consortium, D5.3) while the trial for the PD 1 participant was online, we found that the PD1 has an outstanding participation in social media accounts and she was regularly using educational sites. The system seemed to work and fit very well in PD 1's needs active participation. More specifically, PD 1 reached a high score almost in Social Indicator parameters from the initial stage and improved her score till the end of the trial. More specifically, we found that the PD 1 had an active participation during the whole duration of the trial (20/4/2018–19/5/2018). Regarding the Social Indicator of “Participation and Social Capital” (E-mail, Social media, Fora etc.), we found that the PD1 reached the highest score of 34.79% during the trial, while the worse score in this indicator was 14.29%. As regards to the “Empowerment and Wellbeing” Indicator (YouTube, News, Entertainment, Health etc.), the participant reached the score of 56.89%, since she was visiting the YouTube on a regular basis for cooking recipes. Regarding the “Education and Employment” Indicator (E-learning, pro networks, job search), she reached the score of 25%. This proves that in all Social Indicators, PD 1 had an active participation during the trial.

Success factors

The core factor behind the successful the platform use by this PD participant was the connection of the usage with her very specific interests. What made the difference was that she was able to quickly understand (with the aid of the Persuasive Design elements and her children) how the platform could be used for rewarding experiences relevant to her interests and hobbies.

The implications

This case exemplifies how the platform can forward social inclusion for people at a later stage in life. For older people digital inclusion does not translate into access to resources for employment, education or socialization. Being confined at home, for many hours, these individuals are able to expand the horizons of their interests, and to find more sources for entertainment and fun at home.

**PD 2 participant Case study**

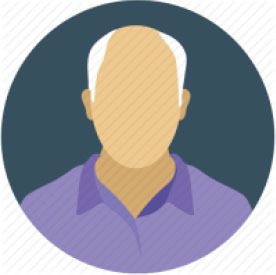

PD 2 is a 53 year's old married man with two teenage children. He is professor of Arts in University and he loves his job. However, he has been diagnosed with PD 11 years ago and since then he has settle back several of his activities, which demand computer use (e.g., e-mail exchange etc.). There are times during the day that he cannot operate simple tasks due to the severe tremor in his upper limbs. He has admitted his clinical condition and he is more than happy to use alternative technological systems in order to feel part of the society and communicate with his students. He spends roughly 5 h a day on the computer/laptop carrying out business tasks, some of them involving extensive writing. His condition impedes his ability to type fast and creates a lot of frustration in him.

His platform usage

He became an enthusiastic user, and he was truly sad to have to stop using it. He was able to learn to use it fairly quickly, and soon enough he was able to be carrying out his digital tasks at a reported faster speed vs. his conventional laptop. He mentioned that the platform enabled him to accomplish more within the time that he allocated to computer use each day. “*I would have to take ages to type an email, and I would make lots of mistakes too, due to my tremor… and the more frustrated I got, the more mistakes I would make. It is very stressful to have to type at much lower speed than your thoughts. The platform made it much easier for me to type fast. And I saw that with each day I was becoming better at using it, too”*

From the data obtained through while the trial for the participant was online, we found that the PD 2 has an outstanding participation in social media accounts and empowerment as well as educational sites. The system seemed to work and fit perfect in PD 2's needs active participation. More specifically, PD 2 reached a high score in several Social Indicator parameters from the initial stage and improved his score till the end of the trial. More specifically, we found that the PD 2 had an active participation during the whole duration of the trial (27/4/2018–26/5/2018). Regarding the Social Indicator of “Participation and Social Capital” (E-mail, Social media, Fora etc.), we found that the PD 2 reached the highest score of 68.14% during the trial, while the worse score in this indicator was 32.14%. As regards to the “Empowerment and Wellbeing” Indicator (YouTube, News, Entertainment, Health etc.) PD 2 participant reached the score of 68.28%. Regarding the “Education and Employment” Indicator (E-learning, pro networks, job search), he reached the score of 48.57%. This proves that in all Social Indicators, PD 2 had an active participation during the trial.

Success factors

For this participant the platform improved quality of life at work, speed and efficiency in daily work related tasks. A secondary benefit of the platform was the easing of the frustration he had been experiencing when using conventional devices with trembling hands. As a result the experience was motivating enough for him to keep making heavy use of it, and become more and more efficient in its use.

The implications

It seems that the platform is able to prove it value best with heavy digital users, who are able to experience dramatically the difference in speed and ease. Even though digital power users are quick to discover the platform merits, we propose that it is the very light digital users who are most excluded from digital social resources, and who need to be encouraged to use the platform and discover its potential value in their life.

In addition to the social and digital inclusion questionnaire, qualitative data were collected from the participants *via* testimonials, open questions as well as *via* interviewer input. In particular 5 out 10 NMD participants state that their symptoms interfere slightly with their ability to use the computer, while 2 out of 10 state that their symptoms do not interfere at all with their ability to use the computer. Their responses to this question reflect their self-perception regarding the use of the computer. It is documented (58, 59) that it is often the case that people with physical impairments tend to reconstruct their perceptions of their everyday life, so that they get a sense of normalcy regardless of their impairment.

When it comes to experiencing the impact of “GazeTheWeb” on social inclusion digital activities, the reaction of the NMD participants is significantly impacted by cognitive dissonance (60), that is, they experience internal inconsistency regarding the role of “GazeTheWeb” in their life. On the one hand they tend to express excitement at using “GazeTheWeb”. In their own words: “*I never thought that there could be a day when I would not need my hands to operate the computer,” “I kind of feel like a pioneer, using a technology that very few people know and use yet”*. On the other hand, they also experience a jolt in their “normalcy reconstruction” process, because “GazeTheWeb” is a strong reminder of their impairment and disability. They enjoy the use of “GazeTheWeb” and its progressiveness, but it also goes against their gut desire to keep their hands active, and to keep using their fingers, as long as they do work. Thus, even though NMD participants understand the significance of “GazeTheWeb” having used it, it is only the NMD participants MDA 5 and MDA 7, with a more severe impairment, who were very resentful at having to part with “GazeTheWeb”, and stated that it made a big difference in their feeling socially included. All of the rest of the participants stated that “GazeTheWeb” would be of critical value to them once their symptoms become a bit more severe. In their own words: “*I have seen how easy and smooth it is to use MAMEM, perhaps I would use it for a bit every day, but I am scared that were I to use ‘GazeTheWeb' every time I use the laptop, then I might be loosing the little wrist and finger movement that I have left faster, for lack of exercise. For the moment, I know I want to keep using the mouse, because this means that I keep exercising my hand, of course it is a big relief to know that ‘GazeTheWeb' is out there available to me, anyway.”* Several of the participants expressed: “*I love MAMEM, it is very exciting to use, and when my situation gets worse, I will be very eager to use it,” “it can be painstaking to use the mouse with my limited hand movement, but I can still use my hand, and this makes me feel like a normal person, but I know ‘GazeTheWeb' will save my life when even this little movement becomes difficult for me.”*

On the other hand, the majority of the SCI participants in the sample find the sitting position tiring. On several occasions a table had to be acquired specifically in order to create a comfortable situation for the use of “GazeTheWeb”. This meant that a new habit, a new routine had to be created for the participant. It is not the case that the participant exchanges one apparatus for another, in an already existing usage environment, as was the case with the NMD and PD participants. Here the trial environment had to be created and this meant an adjustment effort for the participant which may have impacted the social inclusion indicators, given that many of the participants could only have relatively short sessions on MAMEM, getting tired easily in the sitting position. It is very important to note that all of the SCI participants were heavy users of smartphones, and much more so than users of desktop or laptop computers. The SCI sufferers find themselves immobile, but their phone stands in for mobility. It is always attached to them, and moves with them, wherever they are. It is their lifeline in that they depend on it to communicate, to express their needs and to be self-sufficient when they need to search the Internet or to engage in online activities. The most significant and debilitating repercussions of a skeletal injury in the life of victims are three, given the outcomes of this study: (1) Home confinement, which narrows significantly the activity horizons of SCI, and may lead to negative emotions. (2) The loss of a sense of control over their life, something consistently felt by SCI individuals. Several of them have the privilege of 24 h paid support at home, yet the sense of loss of control remains still prevalent. (3) The abrupt severance of life purpose. The majority of the SCI do not have much to look forward to, as on many occasions there is no positive prognosis for their symptoms. They are at a loss as to how to construct meaning and purpose for their life. NMD sufferers focus on delaying the progression of their symptoms, and PD sufferers' focus on enjoying family and home at a later stage of their life. However, SCI individuals in the sample are rather young people who lack a clear positive focus in life, with the exception of SC2, a successful businessman who is able to sustain his business involvement. The big challenge is how to help SCI individuals to sustain a positive life perspective and a sense of inclusion and life productivity.

As regards to PD, “GazeTheWeb” impacted them in varied ways, however there seems to be a common element across all participants and it had to do with the inclusion needs relevant to their life stage (median age: 55 years old). It is not surprising, then, that the Social Capital, Employment and Education indicator scores were low for this sample and that one of the top appreciated elements in “GazeTheWeb” was the ease in watching video and Internet content. “*I have long hours at home with nothing much to do other than watch TV, when I am on the internet it feels like I am not left behind, and it is great to be able to do so many things, browse, and see a bit of shopping, and watch videos.”* In particular, the responses of PD participants mirrored the social stereotypes. Additionally, the learning and adoption process for several of the PD participants was colored by anxiety, that is, the anxiety to perform well in learning the new technology and becoming able to use it at some level of competence. Eventually, the majority of participants preferred to use “GazeTheWeb” for entertainment only, whereas the 1-month duration of the trial proved to be too short for the PD participants.

## Discussion

Despite that 1 month maybe is too short to expect a significant behavioral shift in social inclusion indicators. Our study proved that it was possible for participants with mild to severe motor impairment to use “GazeTheWeb” for a month, and it is reasonable to expect that there can only be indications rather than proof of its social impact given this limited time of usage. People with disability are even slower to adapt to novelty vs. the regular population. For many individuals with disability it is a daily battle to have to adjust to their living conditions, and this slows their adjusting to new elements in their environment. The sense of safety that people with disability develop is fragile and does not leave ample room for novelty. In this context it can be said that “GazeTheWeb” would require longer use for individuals to discover and explore its potential in enhancing their being included in society.

It has been shown in research that it takes at least 2 months of conscientious practice to change automaticities and to adopt new routines and habits. It must be noted that the PD participants in the sample, at their life stage of over 50 years, fulfill the cultural stereotype that wants mature individuals to downshift, to curb their ambitions and to focus more on professional survival than professional achievement (61). Another study describes how embodied negative stereotypes and expectations of aging impact health and functioning of mature individuals (62). At this life stage a cultural stereotype expects people at later life stages to focus less on profession and creating. The cultural stereotype expects them to focus more on connecting with family and grandchildren, rather than expanding their connections beyond family, with the greater community and society. The challenge is how can “GazeTheWeb” capitalize even better the fascination it generates among older age people with disabilities, and be fully used a vehicle that can take people with disabilities at later stages in life into a more socially expansive view of themselves and of their life. The reactions of PD participants brought fully to light the extend of digital exclusion experienced by them, which can be said to be typical of their age group. The indicators of digital exclusion typical of their majority include their basic usage of digital technologies. They mostly use the computer for browsing, and search, and to a lesser extent to watch video content, just as they would watch TV. Their use of social networks, in comparison to the other cohorts, is less extensive. With the exception of two working individuals in the sample, the rest of the PD participants use the computer minimally or not at all to carry out transactions like shopping or e- banking. Their digital exclusion is evident also in their minimal use of a smartphone, contrary to the NMD and SCI participants. It is therefore to be expected that not only would they use “GazeTheWeb” in accordance to their existing habits of computer use which reflect their digital exclusion, but also that their responses to the social inclusion questionnaires would likewise reflect their low involvement with technology. For some of the participants the opportunity to do more with “GazeTheWeb” was an exciting discovery.

On the other hand, the mean age of NMD participants is 31.5 years of age, which is significantly younger than, for example, the mean age of PD participants. Also this younger generation is defined as the digital natives, as they are all “native speakers” of the digital language of computers, video games and the Internet (53). This younger sample has achieved familiarity and ease in their current use of digital devices, despite their disability. Therefore, they are able to appreciate the value of “GazetheWeb”, yet the fact that they are already proficient in the use of their current device makes it very tough for them to discover the dramatic difference in social inclusion indicators that “GazeTheWeb” could bring about in only 1 month of use. We esteem that a longer duration of use might be required for them to reach a level of “GazeTheWeb” mastery, which would make it possible to experience the social inclusion differences in action.

Therefore, the learning and adoption process for several of the older participants was colored by anxiety, that is, the anxiety to perform well in learning the new technology and becoming able to use it at some level of competence. It became clear that older responses felt slightly intimidated by their contact with the progressive technology of “GazeTheWeb”. Though younger people can be intuitive learners of new technologies, older people are digital immigrants who may easily feel vulnerable and less competent in front of new technology. It must be ensured that “GazeTheWeb” offers a completely user-friendly experience especially people at later life stages. According to their research, prevailing social attitudes portray people with disability as dependent rather than autonomous. Wang and Dovidio (63) argue, through their research, that an identity of disability involves a frame of mind that is conducive to caution, restraint, and a sense of helplessness. This is fully corroborated by our findings. It is important to note that when the self-identity of the person is separate from their disability, then their self-perception of what they are able to accomplish changes dramatically. We propose that “GazeTheWeb” is a vehicle that can help the shift in self—identity from one of limitation to one of expansion *via* progressive, advanced, state of the art technology.

Moreover, it was found in this research that people with disability were resistant to social inclusion, and this was more the case with the PD sample and to some extent with the SCI sample as well. According to a recent study (64) people with disabilities experience self-consciousness, especially out of home, and they also internalize cultural stereotypes relevant to their disability and age. In our study, for example, a PD participant was in constant fear of being fired from his sales job because of his PD tremor, and this caused him to be completely intolerant of any mistake he did, even though it took him triple the time to carry out tasks in his conventional computer. Early retirees had internalized stereotypes, which made them think that carrying out productive, or work and skill related tasks on the computer would be useless and not necessary for them. PD participants thought that community and social capital is less important and less relevant to them, as long as they have their immediate family available to them. The challenge for “GazeTheWeb” is to entice individuals with disability into an inspiring and encouraging user experience long enough to win them over to the potential social inclusion. One of the most important findings in the study was the barrier to social inclusion stemming from a debilitated sense of life meaning and life purpose prevalent in many of the participants, regardless of cohort. These individuals struggle to find meaning and purpose in their everyday life and often end up discouraged, on pure survival mode. NMD participants tend to be more of an exception to this, since their condition started at an early enough age, giving them ample time to adjust and find ways for fulfillment despite their disability. Among the NMD sample there was a Paralympic athlete, a full time graphic designer who also participates in theater productions. However, this is not the case with PD and SCI participants whose condition is a source of an extreme sense of vulnerability. We propose that “GazeTheWeb” can be a vehicle opening up horizons of productivity, self-expression and social connection. However, it needs to be accompanied by dedicated efforts to explain and educate the user into the potential of its use.

In particular, the NMD participants exhibited the most favorable reactions patterns with regards to social inclusion, and though there were few statistically significant differences in responses before and after the use of the proposed solution, however this sample exhibited more consistently upward trends in reactions after “GazeTheWeb” usage. This can be explained due to the fact that this is a younger sample of “digital natives”, closer to technology, already using computers fairly heavily. “GazeTheWeb” was for them a fascinating and exciting progressive new technology, which captivated attention and created intention to explore. Moreover, this group suffers from a progressive disease, and knowing that there is a technology that can assist them should they lose complete movement of the hands is a strong motivating element. Indeed, the participants who did not have any movement of the hands were the most enthusiastic adopters of the technology. Therefore, the majority of NMD participants expressed that they would adopt “GazeTheWeb” in the case that they lost movement of the hands completely. When they have a choice between using their hands even with difficulty and big effort, and using a technology that offers complete ease and no use of hands, they consistently choose the use of their hands over ease and convenience. Not using their hands at all gives them a sense of being close to completely disabled. Moreover, they fear that if they stop using their hands, they may lose whatever movement they have left there, because of lack of exercise. Nevertheless, “GazeTheWeb” will have to generate a paradigm shift among the NMD participants. It needs to be able to communicate to them that using “GazeTheWeb” does not mean you stop using your hands, but you are using your mind more and you make your time at the computer count for more. Out of all the social inclusion indicators, the Empowerment indicator exhibited the strongest, statistically significant shift after “GazeTheWeb” use. What was especially impactful among the participants was the very usage of a state of the art, progressive technology, not yet available as a mainstream product, yet accessible to them. NMD participants expressed a sense of accomplishment and self worth out of their month with “GazeTheWeb”. They expressed a strong sense of satisfaction related to mastering a new task and doing so successfully. The type of empowerment experienced could best be described as “a sense of mastery over technology”. In the words of an NMD participant: “*it was like taming a beast, first learning to use ‘GazeTheWeb' and then becoming good at it, and having it do all I wanted, it was a great experience.”* For people with disabilities, who are loosing control over their movement, to be able to have a sense of mastery over technology helps “*making me feel valued,”* and “*making me feel I am playing a useful part in society”*.

With regards to the SCI participants, this groups has been characterized by their physical vulnerability and fatigue when having to use “GazeTheWeb” in the sitting position. The SCI participants are typical of what we might describe as “mobile social inclusion” in the sense that they heavily depend on their mobile phone to express their needs, to connect to their environment and to have a sense of safety. The smart phone is the interface between their sense of helplessness and their world, and it is the vehicle through which they make their environment responsive to their needs. With regards to the social inclusion indicators these participants' overall reactions showed low interest in the employment and education factors, and it became clear that they are resigned and, by now, used to a way of living of curbed options. It was interesting to note that “GazeTheWeb” contributed optimally to the inclusion of the SCI participants when it was used to expand their options, as in online shopping and e banking, which are carried out with less ease on the mobile phone. Being able to shop and carry out tasks on line seemed to give at least some of these participants a stronger sense of control over their lives. At the moment, having to use “GazeTheWeb” in a sitting position is a challenge for many of the SCI participants, since they are prone to fatigue, and prefer the reclining position. Fatigue in the sitting position is by far the major barrier to the use of “GazeTheWeb”. There are two areas that “GazeTheWeb” will need to cater to, in its future evolution, taking into consideration the needs of SCI participants. (1) It needs to technically develop a solution for use in the reclining position and (2) It needs to clearly define its mission and social inclusion benefits vs. the mobile smart phone. Prospective users need to be inspired to understand that they may have a richer interactive and productive online experience with MAMEM carried out with a far improved user experience vs. the smart phone.

Finally, regarding the PD group, given that most of participants were over 55 years old, this sample is characterized by a more introvert attitude and a heavy dependence on family and relatives. Many of them are already retired and the rest of them are pre retirees. Therefore, three main characteristics can be attribute to them: (i) the “digital exclusion”, given that they are not used to using digital devices extensively. They do use them but in a more limited way vs. the other cohorts, (ii) limited expectations regarding social inclusion, given that their attitude reflects the stereotypes for older age groups where ambition for work or for a more extrovert living wear down. The PD participants' world revolves around their family and around old time habits and hobbies. They are not easily receptive to changing their daily routines, perceptions and practices. (iii) They learned “GazeTheWeb” at a lower pace and had lower toleration for the bumps during the learning stage of the technology. Thus, reactions to social inclusion questions were not very encouraging, though some of the participants became excited with the options “GazeTheWeb” offered for an easier and faster use of digital spaces. Overall, it became clear that “GazeTheWeb” would have to be used for much longer than 1 month in order to provide a full experience of its value and benefits. The PD participants who are already won over to the digital world become very excited about using MAMEM, because the PD symptom of tremor makes the use of a computer very difficult and slow. Problems are completely solved with “GazeTheWeb”. The ease of use encourages wider and expansive use of the Internet, deeper involvement with hobbies and interests. Therefore, many of the PD participants do a very basic use of the computer. Using “GazeTheWeb” would mean that they are willing to proceed to a new mindset about how the digital world can enrich their lives. Without this mindset they are prone to feel that “GazeTheWeb” is “… nice to have but not really necessary for quality of life”.

Therefore, with regards to users who are in later life stages, and who are basic users of the digital world, “GazeTheWeb” needs to find ways to inspire them with what is possible and achievable. Easily accessible content (videos, tutorials) needs to be developed showcasing the benefits of “GazeTheWeb”. In general our findings showed positive reactions, above average on a 5-grade Likert scale, across all five of the Social Inclusion Indicator pillars. The Empowerment indicator was the one impacted at a level of statistical significance. In particular the NMD participants experienced a heightened sense of value to society, and an improved sense of choice and freedom, as a result of using “GazeTheWeb”. The challenge for “GazeTheWeb” is reaching a critical improvement level, in their digital experiences, that would offer them expanded inclusion horizons. We esteem that for this to happen completely, a much longer period of use would be required. An important learning is related to NMD being a progressive type of disease. The symptoms are highly probable to progress over time, and the sufferers are aware of this. Holding on to the available hand movement gives them a sense of relief and control, and a sense of holding back the progress of the symptoms by exercising the limbs. Though “GazeTheWeb” would greatly improve their digital experience in terms of ease and speed according to their own feedback, however, many also state that they would adopt it once their symptoms become more severe, and their disability more pronounced. Moreover, our findings showed that physical elements colored responses to “GazeTheWeb” usage: the SCI participants typically tire easily, and especially so in the sitting position. Since “GazeTheWeb” could only be used in a sitting position, some of them were able to use it for relatively shorter sessions than they would desire. Moreover, it was seen in Phase Ii trials that the SCI participants experience digital inclusion through their mobile smartphone, which they carry with them always. The smartphone inspires a sense of connection and security, and constitutes the way in which they make their environment responsive to their needs. It seems that “GazeTheWeb” comes over and above the smart phone to cover their social inclusion needs. The most pronounced effect of “GazeTheWeb” (though not statistically significant) on the SCI users was on the Empowerment indicator questions of “using ‘GazeTheWeb' over time my online activities give me a sense of choice and freedom”. For individuals who experience immobility and dependency the sense of freedom and choice can be a particularly significant and rewarding effect. It is also interesting to note that “GazeTheWeb” did not have a significant effect on the Education and Employment indicators beyond what was already experienced by the SCI users before installing and using “GazeTheWeb” for a month. This is in line with the finding that SCI individuals do not espouse a vision of their future that includes employment or education. Only 4 in the sample of 10 were employed, and of them only one was employed full time. It is important to note that one secondary effect of the spinal injury is the loss of life perspective and life purpose and meaning, and “GazeTheWeb” can acquire a powerful role in the lives of such individuals, to that effect. Finally, the reactions of PD participants to “GazeTheWeb” reflected their relationship with technology, which, at their mature life stage is more limited vs. other younger life stages. PD sufferers tend to be older, and they also tend to be digital immigrants. Digital immigrants are not intuitive assimilators of technology as are the younger digital natives who have evolved skills through years of practice and interaction with technology. When measuring the impact of “GazeTheWeb” on social inclusion indicators we found that some of them were impacted negatively due to performance anxiety and the learning process, given the 1-month period of trial that proves to be too short for this age group. We can hypothesize that the full impact and potential of “GazeTheWeb” would become fully evident once participants became fully efficient and confident in its use. Given the 1-month of trial, the indicator that was impacted most positively was a digital inclusion one “with ‘GazeTheWeb' I was able to watch video and content on line that interests me”. Four elements have been proven in research to influence greatly the assimilation of technology among older age groups. According to a systematic review, these are: (a) anxiety, fear, resistance or cautiousness of using computers; (b) self-efficacy and confidence in the ability to use computers; (c) liking to use computers and digital tools; and (d) the perceived value and usefulness of using digital devices in personal life (65). The PD participants are fully aware of the value and usefulness of digital devices but they lack self-confidence, they experience awe toward new technologies. Thus, the first three of the four elements mentioned above need to be further optimized in the “GazeTheWeb” experience to address the needs of these individuals. Given that the PD suffering, older individuals have distinct and different needs from younger people with disabilities, we propose that a future version of “GazeTheWeb” needs to integrate further persuasive technology elements specifically geared to this age group. These persuasive design elements need to focus more on affirming and rewarding use, rather than motivating usage with competitive devices.

Our study holds some limitations. First of all, we included a small, diverse and heterogeneous sample: Each of the participant samples experienced disability in a different way, and with different psychological repercussions. The three cohorts were at very different levels of connection and usage of the digital world. Moreover, the three cohorts tended to be at different life stages, with different needs. This diversity made it possible to study each cohort individually, but the aggregated data from such a diverse population starts to become somewhat less meaningful and relevant. Indeed an increased sample size would offer more statistical significant differences and would strengther our study. However, this study constitutes a usability study of a certain group of patients after testing the “GazetheWeb”. Although the literature on usability study designs is relatively new, the emerging methodological literature suggests that usability studies should be addressed specifically to descriptively assessing the potential validity of the RCT plan and not to testing the hypotheses of the main RCT (66, 67). Therefore, usability studies are not expected to have the large sample sizes that are needed to adequately power statistical null hypothesis testing. Moreover, presented data that support that observing four to five participants will uncover about 80% of a product's usability problems (66). In 2010, Hwang and Salvendy reported a meta study on the effectiveness of usability evaluation, concluding that a sample size of 10 ± 2 is sufficient for discovering 80% of usability problems (68). Therefore, following the abovementioned suggestions and other similar research approaches we have examined the potential of our suggested solution in 30 participants in total allocated in three different groups of patients (SCI, PD, NMD) and we believe that even in the absence of statistical significant difference, the descriptive statistics of the study raise important about the potential of BCI's in social inclusion of motor-impaired people. Secondly, “GazeTheWeb” was used for a limited amount of time at home: Ideally “GazeTheWeb” should be used for at least 2 or 3 months, but for technical reasons this was not possible or feasible. We propose that just 1 month is not adequate time for users to experience a tractable shift in their social inclusion practices. Further research will be required in the area of how a new technology can captivate user's mindsets, motivations and attitudes, so as to create optimal conditions for creative and constructive change. The next step for “GazeTheWeb” may be to explore ways in which it can generate good will in users toward expanding their up to now digital practices. Given the findings and the diversity of responses across the three cohorts, we propose to approach digital social inclusion on two distinct levels: (i) the supportive social inclusion indicators (provide support, to help reclaim a sense of wellness, and to aid them in connecting back to society and community) and (ii) contributive inclusion indicators (supportive and enabling system in their effort for achievement. People will be enabled to achieve and produce and contribute). The reason to distinguish between two levels of social inclusion is because we found that the needs for social inclusion are not static and solid, but fall on a continuum and “GazeTheWeb” needs to resonate with these needs. Regarding these two social inclusion areas, the challenge for “GazeTheWeb” is to develop its user experience to cater to these distinct needs *via* its integrated persuasive technology, *via* aids like gaming, video, tutorials, demos, testimonials.

Finally, given that this technology offers the promise of greatly enhancing these patients' quality of life by considerably improving their personal autonomy and mobility, “GazetheWeb” could be used as an assistive, adaptive, and rehabilitative technology to monitor the brain activity and translate specific signal features that reflect the elderly's intent into commands that operate any device and further increase their social contact by contacting with other relatives. Therefore, future studies should include also people for instance with Mild Cognitive Impairment due to Alzheimer's disease to increase their social contact resulting in amelioration of both cognitive symptoms but also increasing their emotional status.

## Conclusion

What needs to be noted in terms of the NMD sample and social inclusion is that “GazeTheWeb” needs to face the challenge of potential users resisting to stop the use of the hands for the operation of their digital device, in fear that hand inactivity will speed up the aggravation of symptoms. To tackle this resistance we propose that persuasive technology design needs to be further embedded in the “GazeTheWeb” design, with active suggestions, guidelines, tutorials and activities that will let the NMD sufferers to experience with clarity the future benefits of the system, and how it will maximize their participation and fulfillment out of their daily activities. Previous efforts have shown that expressive disclosure, of the kind that is supported by “GazeTheWeb” has been shown to improve the sense of wellbeing in people motor disorders (69). The potential of “GazeTheWeb” to influence the quality of life of its users is maximized not just by the technology that supports it, but also by the persuasive design evolution that will encourage its use. Finally, people with disability may experience movement impairments, and physical social isolation, yet “GazeTheWeb” intends to facilitate their ability to make choices and decisions in the digital environment, where movement does not play a role, neither impedes one's options and opportunities. As long as the digital environment is readily and easily accessible, and “GazeTheWeb” means to address exactly this issue.

## Data Availability Statement

The raw data supporting the conclusions of this article will be made available by the authors, without undue reservation.

## Ethics Statement

The studies involving human participants were reviewed and approved by Scientific and Ethics Committee of Center and Research and Technology Hellas. The patients/participants provided their written informed consent to participate in this study.

## Author Contributions

SN, IK, IL, AM, and VO contributed to the conception and design of the study. IL, KG, and SN organized the dataset and analyzed the results. IL performed the statistical analysis. IL, KG, SN, and AM wrote the first draft of the manuscript and wrote sections of the manuscript. IK made the final corrections. All authors contributed to manuscript revision, read, and approved the submitted version.

## Funding

This work is part of project MAMEM that has received funding from the European Unions Horizon 2020 Research and Innovation Program Under Grant Agreement No. 644780.

## Conflict of Interest

AM was employed by Muscular Dystrophy Association, Hellas. The remaining authors declare that the research was conducted in the absence of any commercial or financial relationships that could be construed as a potential conflict of interest.

## Publisher's Note

All claims expressed in this article are solely those of the authors and do not necessarily represent those of their affiliated organizations, or those of the publisher, the editors and the reviewers. Any product that may be evaluated in this article, or claim that may be made by its manufacturer, is not guaranteed or endorsed by the publisher.
