# Supplementary material for: Exploring the Connection of Brain Computer Interfaces and Multimedia Use With the Social Integration of People With Various Motor Disabilities: A Questionnaire-Based Usability Study
Source: Front Digit Health. 2022 Aug 4;4:846963. doi: 10.3389/fdgth.2022.846963 (PMC9385967; doi:10.3389/fdgth.2022.846963)
Supplement: Supplementary file 1 [file Data_Sheet_1.docx]

Questionnaires from CRFs

**Overview of the “Before”, “During” and "After" Steps**

| Install monitoring mechanisms one month in advance of MAMEM usage onset | This is especially important for Social Tracker This will ensure that we have enough “before” and “after” data |
| --- | --- |
| Perform the demographic and clinical questionnaires | Can be done over the phone, anytime before the 1^st^ day. |
| Prepare materials for first interview | 1. List of recommended sites to visit 2. List of contact details in case they need help with system operation 3. Card set 4. “Manual” / “How to” |
| Perform the parts of the “Before” interview | 1. Explain MAMEM study 2. Carry out the social inclusion part of the questionnaire 3. Training 4. Carry out the training evaluation questionnaire 5. Encourage social activity and make recommendations 6. Explain audio diaries 7. Ensure person understands troubleshooting steps, provide contact details if they get stuck 8. Explain what comes next |
| Perform the 2-week usage milestone | 1. Telephone call at 2 weeks of usage 2. Evaluate person’s comfort level with MAMEM 3. Check for problems and issues 4. Check how MAMEM compares to their previous device 5. Check whether user is engaging in social activities the same, more or less versus their previous device |
| Perform the parts of the “After” interview | 1. Carry out the social inclusion part of the questionnaire 2. Carry out the QUEST 2.0 and SUS questionnaires |

**Telephone demographic and clinical questionnaires**

**This part of the questionnaire can be carried out via a telephone interview and is directed at new participants only. This information will already be available in the case of participants who have been already involved in previous steps of the study**

**Date performed: __________________________________**

**DEMOGRAPHIC DATA**

| Age |  |
| --- | --- |
| Gender | Male / Female |
| Marital status | Single / married / Divorced / widower/ lives with a partner |
| Number of children |  |
| Ages of children |  |
| Educational years |  |
| Occupation |  |
| If employed/working | Full time / partial |
| Hours employed/working per week |  |
| Hand-use | Left-handed / Right-handed |

**CLINICAL DATA (for SCI participants)**

| Diagnosis: (Neurological level of injury (NLI) & American Spinal Cord Injury association – (ASIA) impairment scale (AIS) | ________________________________ | |
| --- | --- | --- |
| Reason of SCI | Traumatic:   - Sport - Assault - Transport - Fall - Other:   _______________ | Non-traumatic:  ___________________ |
| Years with SCI/year of injury |  | |
| What type of chair do you use for transport? | Motorized/regular | |
| Do you move yourself? | Yes /no | |
| Do you have a car? | Yes / no | |
| If yes, Do you drive? | Yes / no | |
| How many hours per day (approximately) do you spend in bed? | ________________________________ | |
| For how long have you been in a rehabilitation ward / day care ward, if any? |  | |
| Please specify from where you get the financial support (e.g. medical insurance) that you are provided with, in order to address your disease). | _______________________________ | |

In which of the following parts of the body do you present partial or complete paralysis/numbness?

|  | Tongue | Jaw | Neck | Shoulders | Arms | Elbows | Wrists | Fingers |
| --- | --- | --- | --- | --- | --- | --- | --- | --- |
| Complete |  |  |  |  |  |  |  |  |
| Incomplete |  |  |  |  |  |  |  |  |

**CLINICAL DATA (for PD participants)**

- 1. Age at diagnosis ______________________________________________________
  2. Disease duration: _____________________________________________________
  3. H&Y scale ____________________________________________________________

*(Instructions to interviewer: if needed, consult with MD/medical records)*

- 1. Are you in a wheelchair? Yes / No
  2. Are you in bedridden? Yes / No
  3. Have you been in a Vocational rehabilitation Center or program? Yes \ no
  4. If so, please specify what center/program and for how long:

____________________________________________________________________

- 1. Please specify the financial support (e.g. Medical insurance) you are provided with, in order to address your disease).

*(Instructions to interviewer: only name the major sources of income)*

______________________________________________________________

- 1. In which of the following parts of the body do you present partial or complete immobility?

|  | Tongue | Jaw | Neck | Shoulders | Arms | Elbows | Wrists | Hands | Fingers |
| --- | --- | --- | --- | --- | --- | --- | --- | --- | --- |
| Complete |  |  |  |  |  |  |  |  |  |
| Incomplete |  |  |  |  |  |  |  |  |  |

- 1. In which of the following parts of your body do you have tremor?

|  | Tongue | Jaw | Neck | Shoulders | Arms | Elbows | Wrists | Hands | Fingers |
| --- | --- | --- | --- | --- | --- | --- | --- | --- | --- |
| Severe |  |  |  |  |  |  |  |  |  |
| Mild/moderate |  |  |  |  |  |  |  |  |  |

- 1. In which of the following parts of your body do you have dyskinesias (*involuntary movements due to medications)*

|  | Tongue | Jaw | Neck | Shoulders | Arms | Elbows | Wrists | Hands | Fingers |
| --- | --- | --- | --- | --- | --- | --- | --- | --- | --- |
| Severe |  |  |  |  |  |  |  |  |  |
| Mild/moderate |  |  |  |  |  |  |  |  |  |

**CLINICAL DATA (for NMD participants)**

1. Diagnosis (*which kind of NMD your diagnosis is related to*): ____________________

*(Instructions to interviewer: if needed, consult with MD/medical records)*

1. Years since first diagnosis: _______________________________________________
2. Have you had any spinal surgery because of your disease? Yes / No
3. Are you in a wheelchair? Yes / No
4. Are you in bedridden? Yes / No
5. Have you been in a Vocational Rehabilitation Center or program? Yes \ No
6. If so, please specify what center/program and for how long:

________________________________________________________________________

1. Please specify the financial support (e.g. Medical insurance) you are provided with, in order to address your disease).

*(Instructions to interviewer: only name the major sources of income)*

______________________________________________________________

1. In which of the following parts of the body do you present partial or complete immobility?

|  | Tongue | Jaw | Neck | Shoulders | Arms | Elbows | Wrists | Hands | Fingers |
| --- | --- | --- | --- | --- | --- | --- | --- | --- | --- |
| Complete |  |  |  |  |  |  |  |  |  |
| Incomplete |  |  |  |  |  |  |  |  |  |

**Computer use habits**

1. How is your social life affected by your disability?

- My social life is normal.
- There is no significant effect on my social life apart from limiting energetic aspects, such as dancing.
- My social life is restricted and I do not go out as often.
- My social life is restricted to my home.
- I have no social life and feel lonely.

1. Have you any kind of hobby or recreational activity? Yes /No
2. If yes, please specify: _________________________________
3. How is your mobility outdoors affected by your disability?

- I travel frequently for needs / pleasure.
- I travel sometimes.
- I travel very rarely and only when there is an absolute need.
- I cannot travel and must stay home.

1. Of the following systems, which do you own?

- Desktop computer
- Laptop computer
- Tablet
- Smartphone

1. If you own more than one, which one do you use the most? _____________________________________________________
2. Do you use a PC? Yes / No

*(Instructions to interviewer: if the subject does not use a PC – even if he/she owns one - go straight to chapter III.)*

1. If so, how many hours (approximately) a day do you use it? ______________________________________________________
2. How many years of experience do you have using a computer?

______________________________________________________

1. Please indicate your main uses of your computer system and the three most important ones:

(*Instructions to interviewer: can choose more than one; mark an x next to the important three uses*)

| - Social participation (Facebook, forums, etc.) |  |
| --- | --- |
| - Productive activities (writing, editing, etc.) |  |
| - Study (on-line courses, articles, etc.) |  |
| - Games |  |
| - Recreation (movies, music, crossword puzzles, blogs, etc.) |  |
| - Communication (email, Skype, etc.) |  |
| - Activities of daily living (purchases, payments, bank, etc.) |  |
| - Information (Wikipedia, governmental sites, news, maps, etc.) |  |
| - Other: ____________________________________________________________________________________________________________________ |  |

1. Please indicate the main applications you use and the three most important ones:

*(Instructions to interviewer: can choose more than one; if chosen, name the main application the subject use; mark an x next to the important three)*

| - Internet browser: ___________________________________________ |  |
| --- | --- |
| - Email client:________________________________________________ |  |
| - Word processor:____________________________________________ |  |
| - Audio/video/image applications:_______________________________ |  |
| - Spreadsheets (e.g. excel ):____________________________________ |  |
| - Computer games:___________________________________________ |  |
| - Presentation software:_______________________________________ |  |
| - Programming/database:______________________________________ |  |
| - Media editing applications:____________________________________ |  |
| - Other: ____________________________________________________________________________________________________________________ |  |

1. Which operating systems do you work with?

- Microsoft Windows
- Unix / Linux
- Apple MacOS

1. To what extent do your physical symptoms impair your ability to use the computer as extensively and as widely as you might like?

- My symptoms do not interfere at all with my ability to use the computer
- My symptoms interfere a slightly
- My symptoms interfere fairly much
- My symptoms interfere very much
- I am not sure/I do not know

**The social inclusion interview**

The guidelines for the person who will conduct the questionnaire interview are outlined below:

- 1. Introduce yourself fully, explain the scope of the MAMEM project and of the interview.
  2. In conducting the interview with the person with the disability, be sensitive to signs of fatigue, and to whether fatigue causes them to mechanically go through the answers without thinking them through. In that case, it is best to stop and continue the interview at another time.
  3. Go through trouble shooting options: explain who and how can be contacted if participants get stuck, or have problems using MAMEM
  4. Encourage social activity: provide the recommended list of sites, encourage them to use them
  5. Explain audio diaries. Explain the mechanics of how they can use the media player already in their laptop to record their voice. Explain that they are invited to provide at least 3 entries
  6. Describe what comes next: that there will be follow up calls checking on their experience

Explain the objective of the study

It is important for the respondents in this study to fully understand the scope and significance of the MAMEM project, and the social inclusion study. We propose, here, a way to present the MAMEM scope:

*"The objective of this study is to fully understand if and how your use of MAMEM may influence your quality of life, and sense of independence, in terms of social life, hobbies, recreation, information, education and opportunities for employment. Your participation in this study is instrumental in developing a specialized technology like MAMEM, which will assist people with difficulties to use the computer and the Internet with their eyes and mind.*

*This questionnaire will first ask a few questions about your digital habits and life, and then will probe your opinion of MAMEM, given the few hours of exposure you have had to it.
After using it for a month, you will be asked these questions again, in order to see whether and how MAMEM has made it easier and better for you to interact online, and to seek opportunities, resources and information that is important to you.*

*When considering your answers please take into account your regular every day activities. Every one of your opinions is very valuable for this research. So, we will go through the questions one by one, carefully. Should you at any point, need a break, feel free to take it. Your comfort is the priority here. This process is expected to take up about 45 minutes of your time, or less."*

Social inclusion

The social inclusion questions were the same before and after the trials.

After the trials the participants were asked to respond to the questions taking into consideration usage of MAMEM over time.

***Instructions to interviewer: use a card for each of the questions Q1, Q2, Q3, Q4, Q5.*** *The card presents the matrix of responses. Give the card to the interviewee while you are reading each statement. The person can read the answers, while you are asking a series of multiple statements and the answers will not need to be repeated for each statement. This will speed up the interview time. Examples of cards with answers for Questions 2 and 3 follow below:

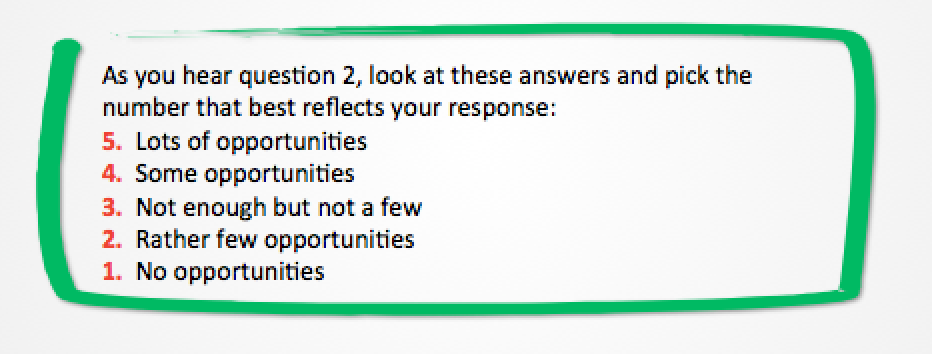
*

**Figure** 1: Example of a response card for Question 2

*
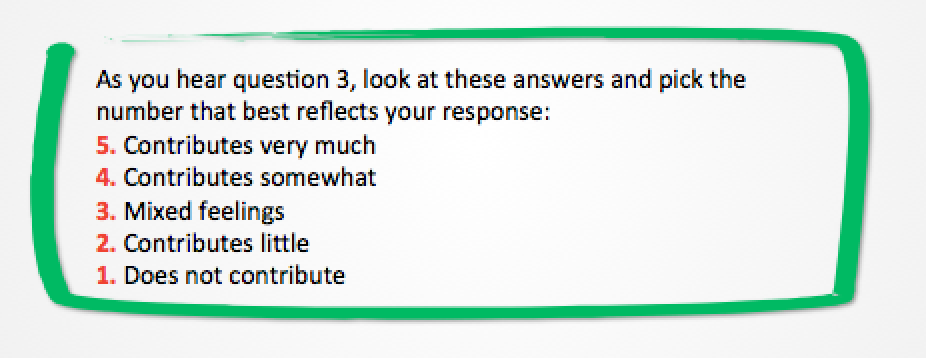
*

**Figure 2**: Example of a response card for Question 3

**Q1**. I will read you some statements that reflect someone’s feelings about life, and I would like you to rate how true they are for you on a scale of 5 to 1, where 5 means the statement is totally true for you and 1 means that they statement is not at all true for you

|  | Very true  for me 5 | Somewhat true 4 | Mixed feelings 3 | Not that true for me 2 | Not at all true for me 1 |
| --- | --- | --- | --- | --- | --- |
| Given my disability, I feel included in most aspects of life around me | 5 | 4 | 3 | 2 | 1 |
| I feel more or less optimistic about the future | 5 | 4 | 3 | 2 | 1 |
| I feel I am playing a useful part in society | 5 | 4 | 3 | 2 | 1 |
| I feel that who I am and what I do is valued by others | 5 | 4 | 3 | 2 | 1 |

**Q2**. I will read you some digital activities and I would like you to tell me how much each of them contributes to your feelings of inclusion in society and ability to make the most of resources available for your benefit. Please on a scale of 5 to 1, where 5 means that digital activities are totally contributing to your feeling of inclusion and 1 means that they are not contributing at all:

|  | Contributes very much 5 | Contributes somewhat  4 | Mixed feelings 3 | Contributes little 2 | Does not contribute  1 |
| --- | --- | --- | --- | --- | --- |
| Active use of digital technologies overall | 5 | 4 | 3 | 2 | 1 |
| Active participation in social media like Facebook, Twitter, Instagram | 5 | 4 | 3 | 2 | 1 |
| Active participation in business, education sites like Linked In, Quora, Academia, etc. | 5 | 4 | 3 | 2 | 1 |
| Attending online courses | 5 | 4 | 3 | 2 | 1 |
| Engaging in online job hunting | 5 | 4 | 3 | 2 | 1 |
| Participating in groups, for a, relevant to your interests and needs (health or otherwise) | 5 | 4 | 3 | 2 | 1 |
| Playing online games with others | 5 | 4 | 3 | 2 | 1 |
| Watching /reading content (videos, movies, books, articles) | 5 | 4 | 3 | 2 | 1 |
| Using specialized software and apps relevant to your hobbies (e.g. photoshop, Picasa, etc.) | 5 | 4 | 3 | 2 | 1 |
| Using digital technologies to earn income | 5 | 4 | 3 | 2 | 1 |
| Hiring help online and finding support on issues that concern you | 5 | 4 | 3 | 2 | 1 |

**Q3**. I will read you some statements now regarding your digital activities and I would like you to rate how true they are for you on a scale of 5 to 1, where 5 means the statement is totally true for you and 1 means not at all true for you

|  | Very true for me 5 | Somewhat true 4 | Mixed  feelings 3 | Not that true for me 2 | Not at all true for me 1 |
| --- | --- | --- | --- | --- | --- |
| There are people online that I trust to support or help me with my problems | 5 | 4 | 3 | 2 | 1 |
| When I go online, there are people I can turn to for advice, about issues or decisions I have to make | 5 | 4 | 3 | 2 | 1 |
| When I feel lonely there are people online that I can connect with | 5 | 4 | 3 | 2 | 1 |
| I engage often enough in digital/online activities that fascinate and entertain me | 5 | 4 | 3 | 2 | 1 |
| My interactions with people online make me want to try new things | 5 | 4 | 3 | 2 | 1 |
| My online activities make me feel a part of a larger community | 5 | 4 | 3 | 2 | 1 |
| I have opportunities to be active and creative through digital / online activities | 5 | 4 | 3 | 2 | 1 |
| My digital/online activities give me a sense of freedom and choice | 5 | 4 | 3 | 2 | 1 |

**Q4**. I will read you some statements now regarding the amount of opportunities you feel you have access to, by engaging in digital and online activities. Please rate the statements from 5 to 1, where 5 means you feel you have access to a lot of opportunities and 1 means there are no opportunities.

|  | Lots of opportunities 5 | Some opportunities  4 | Not enough but not a few opportunities  3 | Rather few opportunities  2 | No opportunities really  1 |
| --- | --- | --- | --- | --- | --- |
| I feel I have access to opportunities to find employment | 5 | 4 | 3 | 2 | 1 |
| I feel I have access to opportunities to acquire new skills | 5 | 4 | 3 | 2 | 1 |
| I feel I have access to opportunities to develop business ideas | 5 | 4 | 3 | 2 | 1 |
| I feel I can pursue promising business contacts | 5 | 4 | 3 | 2 | 1 |
| I feel I can learn more about health issues | 5 | 4 | 3 | 2 | 1 |
| I feel I can communicate and flirt with members of the opposite sex | 5 | 4 | 3 | 2 | 1 |
| I have opportunities to advance my hobbies and my creativity | 5 | 4 | 3 | 2 | 1 |
| I have the opportunity to be a volunteer and to support others meaningfully | 5 | 4 | 3 | 2 | 1 |
